# Computational analysis of eugenol inhibitory activity in lipoxygenase and cyclooxygenase pathways

**DOI:** 10.1038/s41598-020-73203-z

**Published:** 2020-10-01

**Authors:** Francisco das Chagas Pereira de Andrade, Anderson Nogueira Mendes

**Affiliations:** 1 Laboratory of Innovation in Science and Technology – LACITEC, Department of Biophysics and Physiology, Federal University of Piauí, Teresina, Piauí 64049-550 Brazil; 2 Postgraduate Program in Chemistry, Federal University of Piauí, Teresina, Piauí 64049-550 Brazil

**Keywords:** Computational biology and bioinformatics, Cellular signalling networks

## Abstract

Chronic inflammation is triggered by numerous diseases such as osteoarthritis, Crohn's disease and cancer. The control of the pro-inflammatory process can prevent, mitigate and/or inhibit the evolution of these diseases. Therefore, anti-inflammatory drugs have been studied as possible compounds to act in these diseases. This paper proposes a computational analysis of eugenol in relation to aspirin and diclofenac and analyzing the ADMET profile and interactions with COX-2 and 5-LOX enzymes, important enzymes in the signaling pathway of pro-inflammatory processes. Through the analysis of ADMET in silico, it was found that the pharmacokinetic results of eugenol are similar to NSAIDs, such as diclofenac and aspirin. Bioinformatics analysis using coupling tests showed that eugenol can bind to COX-2 and 5-LOX. These results corroborate with different findings in the literature that demonstrate anti-inflammatory activity with less gastric irritation, bleeding and ulcerogenic side effects of eugenol. The results of bioinformatics reinforce studies that try to propose eugenol as an anti-inflammatory compound that can act in the COX-2/5-LOX pathways, replacing some NSAIDs in different diseases.

## Introduction

Eugenol, 2-methoxy-4-prop-2-enylphenol, is an allylbenzene compound, constituent of several plants, such as cloves or basil, described with antiseptic, analgesic and antibacterial properties^1–5^. In view of its potential application in some diseases, this compound has been studied in terms of antioxidant, anti-inflammatory, antispasmodic, antidepressant, antigenotoxic and anticarcinogenic effects^6–8^. The anti-inflammatory characteristics of eugenol still need further studies regarding the signal transduction pathways that are blocked and activated by the immune system. According to the literature, eugenol can inhibit the generation of superoxide anion in neutrophils, by inhibiting the Raf/MEK/ERK1/2/p47phox-phosphorylation pathway^9^. In parallel, aliphenols such as eugenol have been reported as inhibitors of proinflammatory mediators such as, interleukins IL-1 β and IL-6, tumor necrosis factor alpha (TNF-α) and prostaglandin E2 (PGE2), inducible expression of oxide nitric synthase (iNOS) and expression of cyclooxygenase-2 (COX-2), nuclear factor kappa B (NF-κB), leukotriene C4 and 5-lipoxygenase (5-LOX)^9–12^.

Numerous diseases such as osteoarthritis, Crohn's disease, colon cancer, breast cancer and prostate cancer are associated with the progression of chronic inflammation with activation of pro inflammatory mediators such as interleukins and intracellular enzymes such as COX and LOX^12–22^. The inflammatory process is associated with an increase in the production of prostaglandins.

COX is the main enzyme in catalyzing the metabolic conversion of arachidonic acid to prostaglandins, which mediates homeostatic functions in different physiological systems^23–25^. COX-1 is a constitutive isozyme responsible for the basal production of essential PGs during homeostasis^26^. COX-2 is expressed at very low levels under normal conditions^27,28^. However, COX-2 is responsible for the high production of prostaglandins during the inflammatory process and pathogenic stimuli and cancer progress^26–28^.

Lipoxygenases (LOXs) form a heterogeneous class of enzymes that catalyze the peroxidation of polyinstaturated fatty acids^29–31^. The 5-LOX enzyme is a lipoxygenase isoform associated with inflammation, bronchoconstriction, hypersensitivity, anaphylaxis, and asthma^32^. The catalytic activity of 5-LOX is regulated through multiple mechanisms, including Ca^2+^ targeted membrane binding and phosphorylation at specific serine residues^27,33^.

Compounds that have dual COX-2/5-LOX inhibitors can be used in cancer chemotherapy^34^. COX-2 and 5-LOX inhibitors can downregulate the progression of colorectal cancer, reducing the capacity for invasion and proliferation in cells of mouse colorectal cancer cell lines (CT26 cells) and human colorectal cancer (HCA7 cells), through supply of the PI3K/AKT pathway^35^. The combination of celecoxib (COX-2 inhibitor) with MK886 (5-LOX inhibitor) can suppress the growth of pancreatic tumor cells^36^.

The application of drugs or compounds that can act in a double way on COX-2/5-LOX can be promising for the treatment and prevention of cancer by acting on inflammation pathways favorable to the progression of tumors that can be inhibited^34,37–40^. Non-steroidal anti-inflammatory drugs (NSAIDs) are widely used to treat inflammatory symptoms: pain, redness, heat and swelling^32,41^. The activity of NSAIDs is characterized by inhibiting the biotransformation of arachidonic acid (AA), a membrane-bound phospholipid, to prostaglandins (PGs), prostacyclin (PGI2), and thromboxane A2 (TXA2) via cyclooxygenase (COX) enzymes^28,42^. However, Patients under NSAIDs frequently report gastrointestinal inflammation, bleeding, ulcers, in addition to hepatic problems, renal toxicities, and other^43^. NSAIDs also had to add warnings to their labels and limitation of uses in certain patients with risks of cardiovascular complications^44^.

The prospect of new drugs that can act on inflammatory processes and that are safe for treatments is one of the main challenges of drug therapy^32,43^. Within this principle, derivatives of natural products have been used as alternative therapeutic approaches in the search for pharmacological redirection and for the treatment of health problems related to the oxidative stress of inflammatory diseases, atherosclerosis, diabetes, cancer^9^.

Therefore, this paper used computational methodologies to verify the pharmacokinetics of eugenol, as well as its chemical interaction with COX-2 and 5-LOX, comparing it with other nonsteroidal anti-inflammatory drugs (NSAIDs): diclofenac and aspirin. The results suggest that eugenol has interaction with the enzymes COX-2 and 5-LOX. Such a perspective opens the way, highlighting eugenol as a potential NSAID of the COX-2/5-LOX dual pathway and suggesting it as an alternative in the prevention and treatment of cancer.

## Methods

### Protein and ligands structural data

Crystal structures were downloaded as .pdb files from Protein Databank Website (www.rcsb.org). The initial biopolymers were simplified by deleting all ligands and water in the structures using Discovery Studio (version 2016). It was selected and chosen and prepared one of the chains (chein A) of each protein for the docking analyze. The following Crystal structure access codes from Protein Databank (www.rcsb.org) were downloaded and employed for the docking study: COX-1 (3N8W, 3N8X, 3N8Y), COX-2 (3LN1, 1CVU, 4OJT), and 5-LOX (3O8Y, 3V99). The Ligand 3D structures were downloader as .sdf files format from Zinc Library (https://zinc15.docking.org/substances) and optimized by PyMOL (version 2.1.1) and saved as .PDB files. The protein crystal structures were optimized using PyMOL free software and Discovery Studio (version 2016) and saved as .PDB files. All prepare for the molecular docking studies were carried out using AutoDock Vina (version 1.1.2) in CHIMERA (version 1.12).

### Docking

The molecular docking technique was used in order to predict the binding geometry requirements of the target molecules to predict the enzymatic mechanisms interectios os NSAIDs and eugenol wich COX in anti-inflammatory processes and in antitumor activity. Eugenol, diclofenac and aspirin interactions with the COX-1, COX-2, and 5-LOX enzymes was performed by molecular docking, the compound was docked using SwissDock (https://swissdock.ch), a web tool for rapid ligand-based virtual screening of small to unprecedented ultra-large libraries of small molecules^45^. After docking completion, ligand conformations displaying greatest binding affinity and lowest docked energies were chosen and re-docking on Autodock Vina^46^, using Autodock Vina docking protocol^47^. The hydrogen bonds, bond lengths and hydrophobic interactions between enzyme (COX-2) and all ligands were determined by using PyMol. Chimera, PyMol and Discovery Studio programs also ware used for visual inspection and graphical representations of the docking results.

### Docking validation

All the crystalline structures analyzed were obtained by the X-ray diffraction method and presented resolutions smaller than 2.8 Å (Table 1). How validation of the docking process it was performed using Root-Mean-Square-Deviation (RMSD) calculations, calculated using the web-based Dincdocking (https://dinc.kavrakilab.org). RMSD values were calculated by comparing the lowest-energy conformation with each fragment conformation, considering all heavy atoms of the ligand. For the validation of the enticer binding with the amino acid fragments, only connections with up to 3.0 Å of compliance was considered and analyzed. All values and links analyzed and described were exhaustively compared to values and data described in the literature, especially those described and related to the structures searched in the Protein Databank website (www.rcsb.org). After docking completion ligand–protein amino acids fragments interactions in the pocket site was re-docking on AutoDock Vina to the validation of the method.

**Table 1:** Resolution PBD structure.

Macromolecule	Code	Method	Resolution
Cox-1	3N8W	X-ray	2.75 Å
	3N8X	X-ray	2.75 Å
	3N8y	X-ray	2.6 Å
Cox-2	3LN1	X-ray	2.4 Å
	1CVU	X-ray	2.4 Å
	4OJT	X-ray	1.5 Å
Lox-5	3O8Y	X-ray	2.389 Å
	3V99	X-ray	2.252 Å

### Target classes

The study to identify the preferred target classes of eugenol molecules was performed using the SwissTarget (https://www.swisstargetprediction.ch/)^48^ a web tool that aims to predict the most probable protein targets of small molecules^49^ and the data was compared for all the compounds under study and the main target analysed by doking method for each compound.

### PreADMET

The pharmacokinetic and pharmacodynamic properties of ADMET, such as absorption in the human intestine—HIA, plasma protein binding—PPB and blood–brain partition coefficient (log BB) were analyzed by in silico studies by PreADMET (https://preadmet.bmdrc.kr) and SwissADME (https://www.swissadme.ch) online databases to evaluate the pharmacokinetic parameters^50^ to relate drug absorption, metabolism and toxicity^51^ for the drugs and eugenol. Prior to that, SDF (Structure Data File) and SMILES (simplified molecular input line entry system) strings were utilized throughout the genera- tion process. The results were analyzed and compared.

### Dinamics

The geometries and energies of the excited states were calculated using the Time Dependent Density Functional Theory (TDDFT), using IQMol (version 2.11.1) software to perform computational calculations^52,53^. The B3LYP functional was used to predict the structural properties as well as the excitations involving charge transfer^54^. The choice of B3LYP is due to the fact that this base to be considered to be universally functional and presents excellent performance when applied to a variety of systems at a relatively low computational cost^55^.

## Results and discussion

### Evaluation of the preADME profile of the eugenol and NSAIDs (diclofenac and aspirin)

Compared to the NSAIDs, from the druglikeness in silico studies performed (Table 2), eugenol was observed, exposed to drug dispensers, because they were in accordance with the criteria of noncompliance with CMC rule, Rule of Five, and the *leadlike* rule, it may be in accordance with these indices to be qualified as druglikeness compound^56–63^. Eugenol exhibited very similar behavior to diclofenac and aspirin. Molecules that no violate these rules like eugenol don't have problems of bioavailability.

**Table 2:** The druglikeness properties of compounds provided by the preADMET tool.

RULE	Eugenol	Diclofenac	Aspirin
CMC_like	Qualified	Qualified	Qualified
LEAD-like	Suitable	Violated	Suitable
MDDR_like	Mid-structure	Mid-structure	Mid-structure
RULE_of_five	Suitable	Suitable	Suitable
WDI_ like	Out of 90% cutoff	In 90% cutoff	Out of 90% cutoff

The Eugenol violates the MDDR rule in two parameters: (1) the number of ring bonds and (2) as to the number of rotation bonds, due to the number of violations is classified as druglike and nodruglike mean structure^63,64^. Interactions involving aromatic rings are major contributors to protein–ligand recognition and concomitantly to drug design. While that reducing the number of aromatic rings of a molecule might improve its physicochemical properties, such as solubility increasing its bioavailability^63–65^. On the other hand, leadlikes are generally smaller molecules such as eugenol and allow structural incorporations to increase effectiveness during lead optimization and can be incorporated as lead optimization processes^66^. Druglikeness values of the substances are very similar, especially between eugenol and aspirin. Probably because they have more similar chemical structures. These values demonstrate that eugenol has a chemical structure druglikeness.

Table 3 shows a comparison of the pharmacokinetic and physicochemical properties of eugenol, diclofenac and aspirin. Biological compounds that are delivered now, need to cross the intestinal barrier to reach their pharmacological target. Eugenol has better intestinal absorption (HIA) ~ 96.8% than other compounds. The permeability of Caco-2 and MDCK cells is more effective for eugenol when compared to diclofenac and aspirin^67–69^. The substances exhibit many pharmacodynamic similarities. It is worth mentioning the greater capacity of eugenol to cross the blood brain barrier, which is probably related to its analgesic action, as well as its better intestinal absorption and permeability in Caco-2 and MDCK cells.

**Table 3:** Physicochemical properties and ADME values to the substances analyzed by the preADMET and SwissADME.

ID	Eugenol	Diclofenac	Aspirin
BBB—(C.BRAIN/C.BLOOD)	2.25544	1.39652	0.715999
Buffer_solubility (mg/L)	1036.58	687.124	26,109.5
Caco-2 (nm/s)	46.8865	24.5317	20.091
CYP_2C19_inhibition	Inhibitor	Non	Inhibitor
CYP_2C9_ inhibition	Inhibitor	Non	Inhibitor
CYP_2D6_ inhibition	Non	Non	Non
CYP_2D6_substrate	Weakly	Non	Non
CYP_3A4_ inhibition	Non	Non	Non
CYP_3A4_substrate	Non	Non	Non
HIA (%)	96.774447	95.95708	90.17676
MDCK	342.148	51.4637	37.9518
Pgp_inhibition	Non	Inhibitor	Non
Plasma_protein_binding (%)	100	91.95465	37.42363
pure_water_solubility (mg/L)	862.745	6.17953	5844.64
Skin_Permeability	− 1.31092	− 2.57397	− 2,02,531
SKlogD_value	2.66241	3.05106	0.115210
SKlogP_value	2.66241	4.29906	1.36321
SKlogS_buffer	− 2.19978	− 2.63448	− 0.83886
SKlogS_pure	− 2.2795	− 4.68056	− 1.4889
**Physicochemical properties**			
Formula	C_10_H_12_O_2_	C_14_H_11_Cl_2_NO_2_	C_9_H_8_O_4_
Molecular weight	164.20 g/mol	296.15 g/mol	180.16 g/mol
Num. heavy atoms	12	19	13
Num. arom. heavy atoms	6	12	6
Fraction Csp^3^	0.20	0.07	0.11
Num. rotatable bonds	3	4	3
Num. H-bond acceptors	2	2	4
Num. H-bond donors	1	2	1
Molar refractivity	49.06	77.55	44.90
TPSA	29.46 Å^2^	49.33 Å^2^	63.60 Å^2^

**Figure 1:**
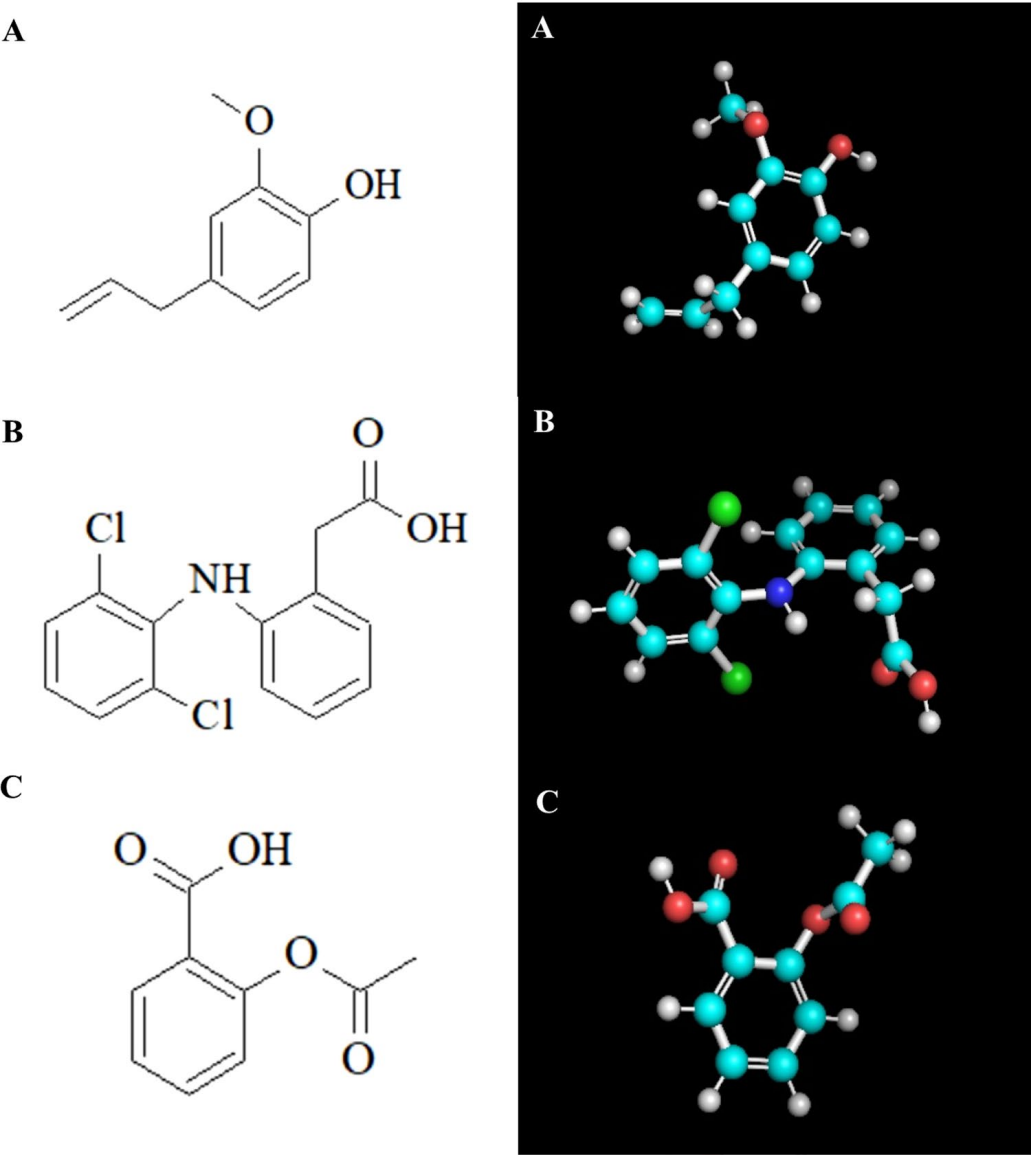
(**A**) Molecular 2D and 3D Structure of Eugenol. (**B**) Molecular Structure 2D and 3D of Diclofenac. (**C**) Molecular Structure 2D and 3D of Aspirin. 2D structure were building in the software ACD Labs ChemSketch release 12.0 (https://www.acdlabs.com/resources/freeware/chemsketch/). 3D structures were downloader from Zinc Library (https://zinc15.docking.org/substances) and saved as .sdf files. The structures were optimised using IQMol software, version 2.11.1 (https://iqmol.org/).

In silico analyzes showed that eugenol has a greater capacity for binding to plasma proteins, justifying a possible plasma transport mechanism and greater capacity for transposing the blood–brain barrier, when compared to diclofenac and aspirin^67–69^. As for solubility in water, eugenol is practically insoluble in water, 5.2 × 10^–3^ mol/L, as well as 6.3 × 10^–3^ mol/L in a buffered solution (pH 7.4). The physicochemical parameters such as SKlogP related to the lipophilicity of chemical substances show the compound is quite liposoluble^70–72^. That indicates the eugenol availability in the plasma membrane when absorbed by the organism (Table 3 and Supplement 1). The structural similarity between the molecules of the substances is evidenced especially between eugenol and aspirin especially by the comparison between the number of rotatable bonds, H-bond acceptors number, heavy atoms number and molecular mass (Fig. 1).

In silico toxicity tests (Table 4) predicted similar toxicity for the Ames test for the tree compounds. For TA100 and TA1535 two strains of Salmonella typhimurium that are frequently used in the Ames test containing the same base pair replacement mutation hisG46^73–75^, eugenol exhibit aspirin-like behavior. The model predicted positive carcinogenicity results for eugenol in both rats and mice, differing from the other two compounds that show negative results, except for aspirin in rat tests. All the compounds under study also present a medium risk for inhibition of the hERG gene, encoding the rectified potassium channel of the rectified voltage into the heart (IKr) involved in cardiac repolarization. The results point to the insured use of eugenol when compared to the other drugs analysed. The substances exhibit many toxicological similarities. This similarity is even greater between eugenol and aspirin. Probably because they have more similar chemical structures.

**Table 4:** Prediction values of the substances analyzed by the preADMET web-based tool.

ID	Eugenol	Diclofenac	Aspirin
Algae_at (mg/L)	0.0567231	0.0194363	0.136893
Ames_test	Mutagen	Mutagen	Mutagen
Carcino_mouse	Positive	Negative	Negative
Carcino_rat	Positive	Negative	Positive
Daphnia_at (mg/L)	0.118703	0.0307894	0.611196
hERG_inhibition	Medium_risk	Medium_risk	Low_risk
medaka_at (mg/L)	0.0188822	0.00181984	0.452842
minnow_at (mg/L)	0.0124586	0.00135885	0.230825
TA100_10RLI	Positive	Negative	Positive
TA100_NA	Positive	Negative	Positive
TA1535_10RLI	Positive	Negative	Positive
TA1535_NA	Positive	Negative	Positive

COX-2-specific NSAIDs are weak organic acids, and lipophilic^24,28,41,76^. Thus, the lower the pH, the greater is their lipophilicity like eugenol. This combination of chemical properties allows that similar NSAIDs COX-2-specific compounds like eugenol (as well as conventional NSAIDs) to cross lipid membranes, including the blood–brain barrier, and to accumulate in acidic tissues such as the stomach, renal medulla, and sites of inflammation^77^.

### Evaluation of the docking of COX with eugenol and NSAIDs (diclofenac and aspirin)

**Figure 2:**
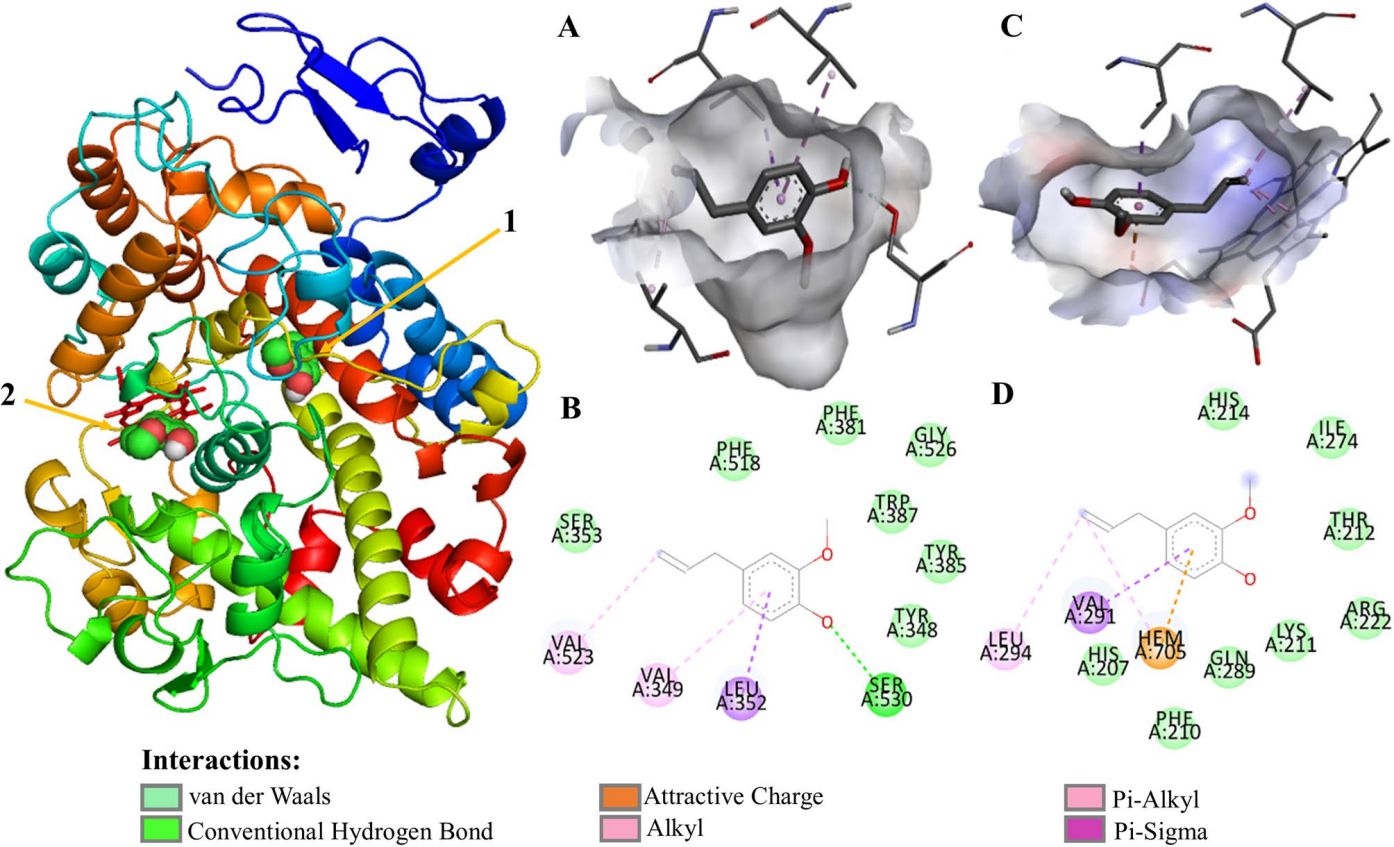
Eugenol molecular docking and molecular interaction with COX-2 active pocket of. (**A**) Eugenol alignment with COX-2 hydrophobic pocket; (**B**) molecular interaction of eugenol with COX-2 active pocket fragments; (**C**) eugenol alignment with COX-2 heme pocket; (**D**) molecular interaction of eugenol with COX-2 heme pocket fragments. The dashed lines in yellow evidence the hydrophobic interactions; the green dashed lines the interactions by hydrogen bonds. The COX-2@Eugenol complex structure were generated in PyMOL version 2.1.1 (https://pymol.org); the structures (**A**–**D**) were generated in Discovery Studio software version 2016 (https://bioviaonline.com/).

COX isoforms have two active sites, cyclooxygenase and heme-dependent peroxidase, which catalyze the conversion of AA to prostaglandin G2 (PGG2) and the conversion thereof to PGH2, respectively (Figs. 2, Supplements 2, 3). On the opposite side of the protein from the membrane-binding domain, the peroxidase active site consists of the heme positioned at the bottom of a shallow cleft^78^. The structural modification results of an exchange of valine at the position of 523 in COX- 2 for relatively bulky isoleucine (Ile) residue in COX-1 at the same position of the active site of the enzyme^41,79^ resulting in a cavit there is not observed in COX-1 that results in steric hindrance at the active site of the enzyme, constraining the valine fragment in COX-2, which is much less bulky. This larger opening of the active site of COX-2 allows greater accessibility of larger molecules that could not interact with the active site of the enzyme. Which makes the COX-2 isozyme active site (volume = 394 Å^3^) is about 25% larger than the COX-1 isozyme-binding site (volume = 316 Å^3^).

**Figure 3:**
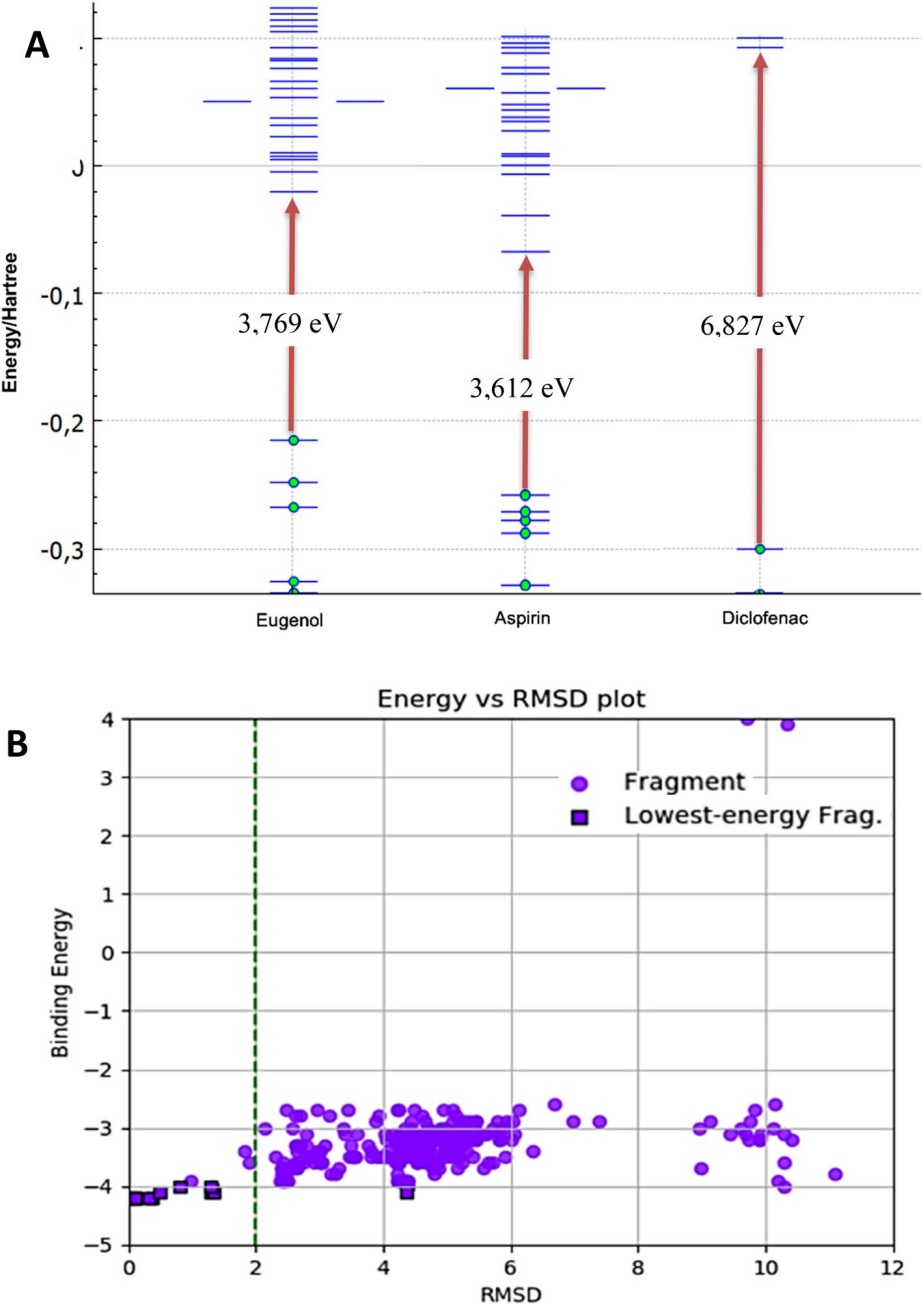
(**A**) Gap of the highest occupied orbital (HOMO), and lowest molecular orbital (LUMO) structure computed using DFT method of eugenol, aspirin and diclofenac. In the grafic the reference values are shown in Hartree units, but values in electro-volts were also calculated for each compound and shown in evidence. (**B**) RMSD for eugenol interactions with COX-2. The purple circles represent the different spatial configurations of the ligand; the purple squares with black edges the lower energy settings. The vertical dashed green lines indicate the ideal RMSD values (values < 2).

There are several common features between COX-1 and COX-2 active sites, the main hydrophobic channel, the catalytic Ser-530 and the mouth having polar residues such as Arg-120. However, COX-1 is characterized by the absence of the side pocket and by a narrower main hydrophobic channel^44^. The COX-2 active pocket (Table 5, Supplement 2) consisted of nonpolar amino acid residues Alanine (Ala 527), Valine (Val 523, Val 349, Val 344, Val 116), Phenylalanine (Phe 518, Phe 381, Phe 209, Phe 205), Leucine (Leu 534, Leu 531, Leu 384, Leu 359, Leu 352, Leu 117), Isoleucine (Ile 345), Methionine(Met 535, Met 522, Met 113), Triptófano (Trp 387), Methionine (Met 508); and polar amino acid residues Serine (Ser 530, Ser 353), Tyrosine (Tyr 385, Tyr 355, Tyr 348), Glycine (Gly 526), and basic fragment Arginine (Arg 120)^80^.

**Table 5:** Interaction types and amino acids involved in the inhibition of COX-2 cyclooxygenase catalytic (PDB ID: 4OTJ) with NSAIDs and eugenol.

Name	Hydrogen bond (HB) interaction	Bond length (Å) for HB interaction	Hydro-phobic interaction	Pi-sigma/Pi-sulfur/Pi-amide interaction	Pi–Alkyl/Pi–Aryl Interaction	Pi–Pi T-shaped interaction
Arachidonic acid	Ser 530 Tyr 385	1.8 1.9	Arg 120 Gly 526 Leu 384 Leu 359 Leu 352 Met 113 Met 522 Phe 518 Phe 381 Phe 209 Phe 205 Ser 353 Thr 206 Trp 387 Tyr 355 Tyr 348 Tyr 206 Val 523 Val 349 Val 344 Val 116	–	Ile 345 Leu 531 Leu 117 Met 535	–
Diclofenac	Ser 530 Tyr 385	1.8 1.9	Gly 526 Met 522 Phe 381 Phe 205 Ser 353 Tyr 355 Tyr 385 Tyr 348	Val 349 (Pi-sigma)	Ala 527 Leu 352 Val 523	Trp 387 Phe 518
Aspirin	Ser 530 Val 523	2.1 2.1	Ala 527 Glu 524 Leu 384 Leu 352 Phe 381 Phe 518 Tyr 348 Val 349	Gly 526 (Pi-amid) Met 522 (Pi-sulfur)	–	Trp 385 Try 385
Eugenol	Ser 530	2.2	Gly 526 Phe 518 Phe 381 Ser 353 Trp 387 Tyr 385 Tyr 348	Leu 352 (Pi-sigma)	Val 523 Val 349	–

COX-2 selective inhibitors explicitly bind to this secondary-binding active site pocket lined resulting in the specific inhibition of COX-2 activity. In a recent mutational study described the involvement of hydrophobic pocket residues in the proper positioning of fatty acid substrates for oxygenation evidencing that highly potent and selective COX-2 inhibitors should possess a pharmacophore which can selectively bind in the secondary pocket and deliver sufficient steric bulk to block the hydrophobic channel of COX-2^81^. Diclofenac (2-[2-(2,6-dichloroanilino) phenyl] acetic acid) is NSAIDs COX-2 non selective inhibitor employed for the treatment of inflammatory symptoms (pain, redness, heat, and swelling) by means of block the formation of prostaglandins resulting in the reduction of acute and chronic inflammation^82–85^.

The computational data showed (Supplement 2B,F) the docking of diclofenac in the COX-2 shows interactions by H-bond with the cyclooxygenase active pocket catalytic residues Tyr 385 Ser 530 and 385; π–π T-shaped interactions between Phe 518, Trp 387 residues and non-chlorinated aromatic ring. There were observed alkyl and π-alkyl bonds between diclofenac molecule and Val 523, Val 349, Ala 527, Leu 352 amino acid fragments, interactions there is interaction of π-electron cloud over an aromatic group and electron group of any alkyl group. The residues Tyr 355, Tyr 348, Ser 353, Phe381, Phe 205, Gly 526 and Met 522 interact with diclofenac molecule by Van der Walls forces. All these interactions allow the diclofenac molecule to act by blocking the canal by an esoteric hindrance, causing its narrowing to impede the access of arachidonic acid to the active site where its catalytic conversion to prostaglandin occurs.

Aspirin (2-Acetoxybenzoic acid) is another nonsteroidal anti-inflammatory drugs target the cyclooxygenase enzymes (COX-1 and COX-2) to block the formation of prostaglandins resulting in the reduction of acute and chronic inflammation^86,87^. Similarly to the data found for diclofenac, aspirin (Supplement 2C,G) demonstrates its action by blocking the hydrophobic channel in the cyclooxygenase active pocket. The molecular docking of aspirin in the COX-2 shows interactions by H-bond with the cyclooxygenase active pocket catalytic residues Ser 530 and Tyr 385. Also there were observed π–π T-shaped interactions between the molecule and Tyr 385 and Trp 387 fragments of COX-2. As well π-amid whith Gly 526 and π-sulfer whith Met 522. The fragments Ala 527, Glu 524, Phe 381, Phe 518, Leu 352, Leu 384, Tyr 348 and val 349 522 interact by Van der Walls forces with aspirin. However, aspirin also inhibits the conversion of prostaglandin G2 peroxidase to prostaglandin H2 at the active site of the heme group (Supplement 3B,E).

Aspirin binds to the Ser-530 site by covalent interaction by acetylating the enzymes cyclooxygenases^88^. This acetylation can inactivate COX-1 or induce COX-2 to produce 15R-HETE (15R-hydroxyeicosatetraenoic acid)^88,89^.

Eugenol similarly to diclofenac and aspirin blocks the cyclooxygenase hydrophobic channel of the enzyme by means of hydrophobic H-bonds interactions with the fragment Ser 530, and hydrophobics interactions with Tyr 385. Eugenol, diclofenac and aspirin demonstrates the ability to interact strongly with the amino acid Ser-530, and the mouth has polar residues like Tyr 385 through hydrophobic interactions. Ser-530 and Tyr-385 are important for the inhibition of COX-2 by several compounds besides aspirin^90^. Ser-530 has also been shown to influence the stereochemistry for the addition of oxygen to the prostaglandin product. The catalytic residue Tyr385 is responsible for the conversion of AA to prostaglandin G2 by the transfer of an electron to the heme from Tyr-385 of the protein generates to tyrosyl radical in the cyclooxygenase active site.

Eugenol has also been shown to inhibit arachidonic acid catalysis in prostaglandin G2 directly in the active pocket site at the end of the hydrophobic channel of the COX by H-bond interaction with Ser 530. In the same pocket interact by hydrophobic forces with the fragments Ser 353, Phe 381, Phe 518, Gly 526, Trp 387, Tyr 348, Tyr 385, alkyl interaction with Val 349, π-alkyl with Val 523 and π-sigma with Leu 352 (Fig. 2 and Table 5).

Diclofenac, aspirin and eugenol has been shown to inhibit arachidonic acid catalysis in prostaglandin G2 directly in the active pocket site at the end of the hydrophobic channel of the COX by polar interaction with Ser 530 (Supplement 2D,H). Inhibition of the active binding site can result in specific inhibition of COX-2 activity (Supplement 3C,F).

Charges of atoms (eV), distribution of relative electron density, vibration energy, localization and energy of highest occupied molecular orbital (HOMO) and lowest unoccupied molecular orbital (LUMO) Hartree Energy, molecular electronic density are very strong similarity between those molecules (Supplement 4) particularly the gap energies HOMO–LUMO between eugenol and aspirin (3,769 eV e 3,612 eV respectively) as shown in Figs. 3A. Those molecules also have a very close surface area, eugenol has a surface area of 388,859 Å^2^ and aspirin 379,426 Å^2^. That explain them similar behaviour and interactions in the COX-2 hydrophobic active pocket.

Eugenol also occupies an orientation very similar to the binding mode of arachidonic acid in the COX-2 active site. The computational results evidence this similar orientation (Supplements 2, 3). According to the computational analysis, eugenol interacts with regions of the active site of COX-2 into the hydrophobic pocket. Ser 530 and Tyr 385 are the key amino-acid fragments that contribute considerably in the inhibition of the protein by the interaction protein–ligand of the active pocket of the enzyme.

Eugenol has also been shown to act on the inhibition of the active site in which peroxidase is responsible for converting prostaglandin to G2 into prostaglandin H2 by the interaction with the group with the lateral methyl radicals of the pyrrole rings of the heme group. The compound also acts on the inhibition of prostaglandin G2 peroxidation in prostaglandin H2 interacting with the carbonic chain of the branching of the pyrrole rings that present the carboxyl function by salt bridge whit hem group, salt bridges contribute little to protein stability but, can make crucial contributions to ligand–protein binding stability. Eugenol also interacts with peroxidase active pocket fragments by van der Waals interaction with Ala 274, Arg 222, Gln 289, His 2014, His 207, Ile 274, Lys 211 and Thr 212; Leu 294 π-alkyl interactions and π-sigma whit Val 291 (Fig. 2).

The peroxidase site presents a heme group like cofactor. The heme cofactor is not bound covalently to PGHS and there are relatively few protein-heme interactions by Van der Waals interactions. The heme iron is coordinated on the proximal side with a nitrogen of a His 207. The coordination with a His 207 is conserved across the heme dependent peroxidases, however, in this peroxidase, the bond length is much longer than normal. A relationship between heme-nitrogen bond suggesting that interactions on the distal face of the heme affect the proximal bond length and reduction potential of PGHS.

The interaction of the binders with the structures obtained in the PDB found by the algorithm was analyzed using the Root-Mean-Square-Deviation (RMSD) calculation, which generally measures the degree of similarity between the structures. RMSD values < 2.0 Å (Fig. 3B) and the low ΔG binding values (Tables 6 and 7) indicate topological similarity between structure and the cavity in the active site and evidence the binding capacity of the ligand in COX-2.

**Table 6:** Compounds docking bind energy.

Protein	Active pocket	∆G (kcal/mol)				
		Arachdonic acid	Eugenol	Aspirin	Diclofenac	Celecobix
COX-2	Cyclooxygenase	− 9.74	− 6.69	− 7.53	− 8.08	11.3
	Peroxidase	− 8.82	− 6.87	− 7.45	–	–
5-LOX	Heme pocket	− 6.00	− 6.59	–	–	–
	Hydrophobic pocket	− 5.10	− 6.02	–	–	–

**Table 7:** Eugenol docking pocket site binding energy in COX-1/COX-2 and 5-LOX.

Protein	Active pocket	Active pocket marker	∆G (kcal/mol)	Ki (µM)
COX-1	Cyclooxygenase	Ser 530	− 6.20	28.3
	Peroxidase	–	–	–
COX-2	Cyclooxygenase	Ser 530	− 6.69	12.4
	Peroxidase	His 207	− 6.87	9.14
5-LOX	Heme pocket	His 367, 372	− 6.59	14.67
	Hydrophobic pocket	Val 234	− 6.02	38.4

Both the cyclooxygenase and the peroxidase active sites are located in the catalytic domain of the COX-1/2 isoforms. Docking analysis of eugenol with the COX-1/2 isoforms (Table 6) shows the eugenol's ability to interact with both isoforms, however a slightly larger docking binding energy module of the protein–ligand complex is observed with the COX-2 cyclooxygenase (− 6.69 kcal/mol) compared to the complex formed with COX-1 (− 6.20 kcal/mol). Eugenol also have showed the ability to interact with the active peroxidase site (− 6.87 kcal/mol), and present a higher inhibition constant against the active site of peroxidase. The large number of NSAIDs and COX-2 selective inhibitors bind in cyclooxygenase active site but not in the peroxidase site^91^. Only Few COX inhibitors have been capacity against the peroxidase activity. Inhibited COX-2 cyclooxygenase, for example, in still capable to accommodate large hydroperoxide substrates such as PGG2^92^. The mean binding energies of the ligands with COX-2 are fairly close values. However, eugenol showed potential to inhibits both active sites of COX-2, increasing the potential of enzymatic inhibition to COX-2 activity of the eugenol wich a total docnkin complex energy of -13.56 kcal/mol highest the docking energy of Celecobix, a selective COX-2 inhibitor.

The binding forces of the compounds with COX-2 are also very close among the analyzed substances (Table 6). Arachidonic acid has a higher binding energy with the Heme group and the opposite side. However, eugenol has binding energy very close to aspirin. These results suggest that eugenol may interact with the Heme group and the opposite side, inhibiting the COX-2 enzyme, similarly to aspirin. In parallel, the weaker interactions between eugenol and COX-2 suggest that the inhibitory action can be reversed. This possibility can be used for studies that involve reversing side effects caused by continuous COX-2 inhibition in chronic patients who need to make continuous use of NSAIDs.

Table 7 shows the interaction of eugenol with 5-LOX binding sites. The binding energy of eugenol with the active sites of 5-LOX, suggests the interaction between eugenol and 5-LOX. This factor may be an indication for COX-2/5-LOX double inhibition activity. According to the literature, there is evidence of the inhibitory activity of eugenol in relation to the enzyme 5-LOX^93,94^. Dual inhibition of COX-2 and 5-LOX enzymes of the arachidonic pathway is a evidence to the capable of the eugenol as anti-inflammatory agent action^95^. In vitro studies suggest that eugenol inhibits 5-LOX by a non-competitive mechanism, decreasing the production of C4 leukotriene in Polymorphonuclear leukocytes^94^. Therefore, the computational results of the molecular interaction of eugenol with 5-LOX reinforce the evidence from in vitro studies.

Dual COX-2/5-LOX inhibitors have the advantages of enhanced anti-inflammatory potency with better safety profile. Whereas the traditional NSAIDs, like aspirin and diclofenac, inhibit cyclooxygenase pathway non-selectively and produce gastric mucosal iritation and ‘Coxibs’ which are selective COX-2 inhibitors, cause adverse cardiovascular events^37^. 5-LOX/COX double inhibitors have been the potential to safety and efficacy to treat inflammation processes, blocking the formation of the prostaglandins and leukotrienes^92^, and could provide numerous therapeutic advantages in terms of anti-inflammatory activity, improved gastric protection and safer cardiovascular profile compared to conventional NSAIDs^96^.

Due to the greater widening of the hydrophobic channel of COX-2 to COX-1 by the replacement of isoleucine 523 by another less voluminous vanillin, more voluminous ligands appear to be more selective in inhibiting the catalytic action by the enzyme, since they block more the active site of the enzyme. However, despite the similar volume, eugenol could will be an important lead for the development of new selective COX-2 drugs.

### Evaluation of the docking of dual COX1/2–5LOX inhibition with eugenol and NSAIDs (diclofenac and aspirin)

In the active site of 5-LOX there are three histidines coordinated to the nonheme catalytic ion Fe^3+^ (His 367, His 372 and His 550), plus an isoleucine (Ila 673) and a valine (Val 671). In addition to the iron attachment pocket, a substrate attachment slot contains several hydrophobic residues and additional amino acids that of LOX inhibitors into the pocket. Arachdonic Acid interact in 5-LOX active pocket with the fragments Arg 246, Val 361, Ala 453, Val 243, Leu 244.

**Figure 4:**
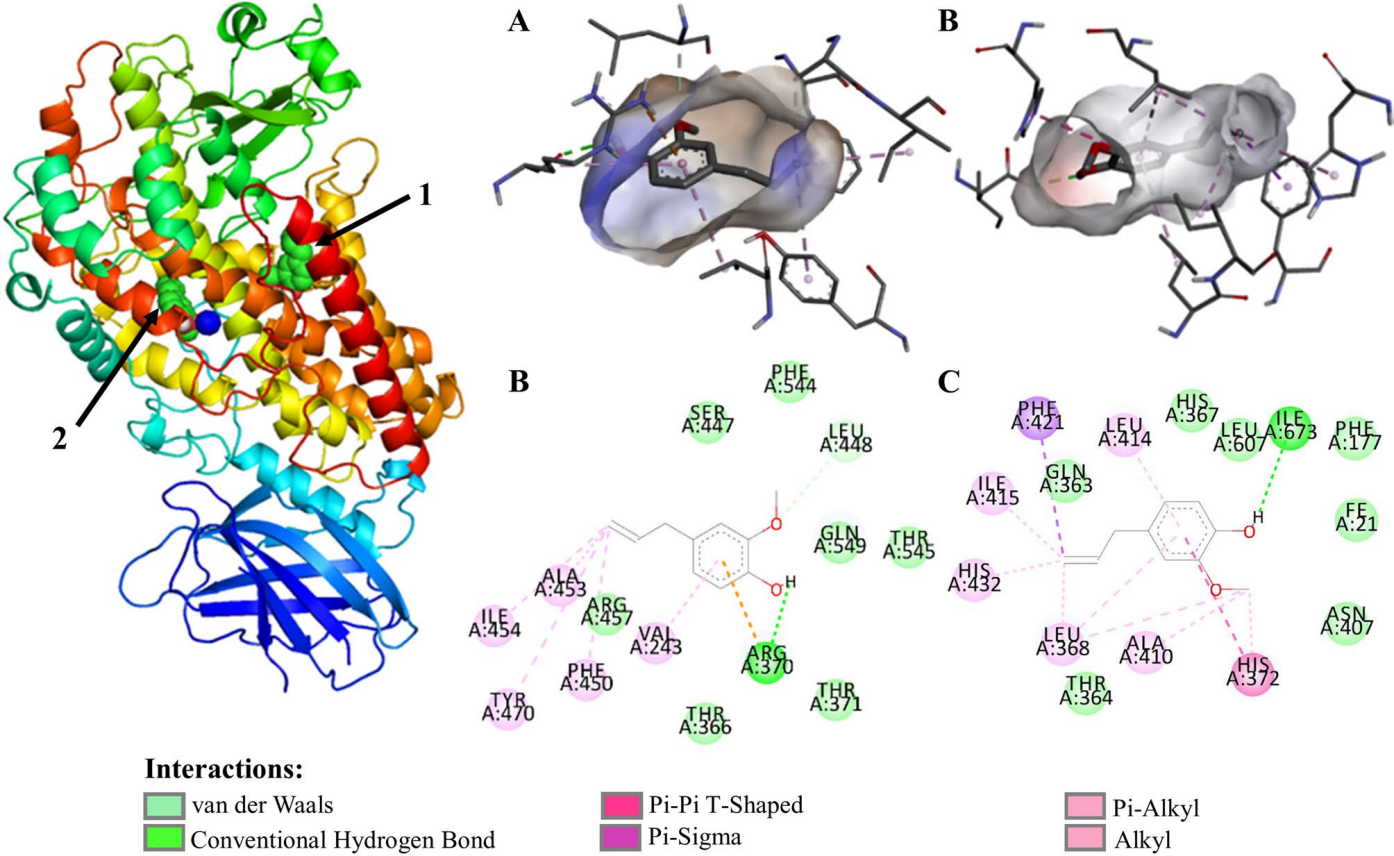
Eugenol molecular docking and molecular interaction with 5-LOX active pocket of. (**A**) Eugenol alignment with 5-LOX hydrophobic pocket; (**B**) molecular interaction of eugenol with 5-LOX hydrophobic binding pocket; (**C**) eugenol alignment with binding pocket of the 5-LOX with coordinated histidine-Fe (III); (**D**) molecular interaction of eugenol in binding pocket of the 5-LOX with coordinated histidine-Fe (III); The dashed lines in yellow evidence the hydrophobic interactions; the green dashed lines the interactions by hydrogen bonds. The 5-LOX@Eugenol complex structure were generated in PyMOL version 2.1.1 (https://pymol.org); the structures A, B, C and D ware generated in Discovery Studio software version 2016 (https://bioviaonline.com/).

Docking studies revealed that the eugenol interacts in the active site of COX-2 as well as with 5-LOX. Eugenol had shown better interaction stabilized of with the active site amino acids of 5-LOX targets respectively the fragments coordinated with Fe^3+^ His 367 by van der Waals interactions, His 372 by π-π T-shaped, and Ile 673 by H-bonds besides other fragments close to the group coordinated to iron ion as Thr 364 by van der Waals interactions (Fig. 4 and Supplement 5). In the additional pocket of 5-LOX eugenol interact whit Val 243, Ala 453 by hydrophobic interactions (alkyl and π-alkyl) and by hydrogen bond and electrostatic interaction between eugenol and Arg 370 (Supplement 6). In π–π T-shaped interaction π–electron cloud between the aromatic groups of amino acid fragments and non-chlorinated aromatic ring on diclofenac in a T-shaped manner, i.e., sidewise electron cloud of the ring and head on electron cloud of other ring, these bonds/interactions are necessary to have temporary interactions, especially for the drug action to be accomplished in a system, in addition interactions involving aromatic rings are major contributors to protein–ligand recognition and concomitantly to drug design.

Dual COX-2/5-LOX inhibitors had demonstrated excellent analgesic and anti-inflammatory activities with lower gastric irritation, bleeding and ulcerogenic side effects, and are an interesting alternative to provide safer NSAIDs^30,37,43,97–99^. The development of drugs with dual inhibitory activity for COX-2/5-LOX enzymatic pathways offers new options for the development of more effective anti-inflammatory agents with an improved safety profile.

Study in vitro and in vivo models on the cyclooxygenase and lipoxygenase pathways to confirm the results presented, are still necessary. However, computational studies can optimize understanding of how a molecule can act on signaling pathways. Therefore, the computational results can help in elucidating the signal transduction pathways that explain the antiseptic, anesthetic and anti-inflammatory properties of eugenol. The possibility of eugenol acting as an dual inhibitory activity for COX-2/5-LOX enzymatic pathways may shed light on new drugs with structures similar to eugenol that can act in chronic inflammation and control of diseases such as cancer, arthritis, autoimmune, cardiovascular and neurological diseases.

## Conclusion

The results of molecular prediction for absorption, distribution, metabolism, excretion and toxicity show that eugenol can be used as a pharmacological compound that has similarities to diclofenac and aspirin. Molecular coupling revealed mechanisms of interaction of the eugenol molecule with amino acids at the active sites of COX-2 and 5-LOX. Molecular modeling suggests which regions of amino acids eugenol can act in inhibiting both COX-2 and 5-LOX, being a relevant way to understand the mechanism of inhibition of enzyme activity, indicating the mechanism that eugenol must act physiologically. The possibility of molecular fitting of eugenol with dual activity of COX-2/5-LOX, demonstrating its potential as an anti-inflammatory agent to act in the composition or synthesis of new selective drugs to fight diseases that need to inhibit inflammatory processes such as: osteoarthritis, Crohn's disease and cancer.

## Supplementary information

Supplementary Information.
