# Supplementary material for: Computational analysis of eugenol inhibitory activity in lipoxygenase and cyclooxygenase pathways
Source: Sci Rep. 2020 Oct 1;10:16204. doi: 10.1038/s41598-020-73203-z (PMC7530671; doi:10.1038/s41598-020-73203-z)
Supplement: Supplementary file 1 — Supplementary Information. [file 41598_2020_73203_MOESM1_ESM.docx]

**SUPPLEMENTARY MATERIAL**

**COMPUTATIONAL ANALYSIS OF EUGENOL INHIBITORY ACTIVITY IN LIPOXYGENASE AND CYCLOXYGENASE PATHWAYS.**

Francisco das Chagas Pereira de Andrade^1,2^ - ORCID: 0000-0003-0141-2341

Anderson Nogueira Mendes^1,2*^- ORCID: 0000-0002-9778-3667

1. Laboratory of Innovation in Science and Technology – LACITEC, Department of Biophysics and Physiology, Federal University of Piauí, 64049-550, Teresina, Piauí, Brazil
2. Postgraduate Program in Chemistry, Federal University of Piauí, 64049-550, Teresina, Piauí, Brazil.

*Corresponding author at: Dr. Anderson Nogueira Mendes, Laboratory of Innovation in Science and Technology – LACITEC, Department of Biophysics and Physiology, Federal University of Piauí, Teresina, Piauí, PI, Brazil phone number: +55 86 32372105, e-mail address: [anderson.mendes@ufpi.edu.](mailto:anderson.mendes@ufpi.edu.)br

**Supplement 1. Druglikeness Prediction of Eugenol.**

(A) Eugenol Druglikeness Prediction, the pink area represents the optimal range for each properties (B) Boiled-Egg Eugenol Druglikeness Prediction. The compound demonstrates low solubility in water due to its low polar character, few rotational bonds and therefore low flexibility and few isoforms due to the self-number of unsaturations, Csp^2^ bonds of the aromatic ring and propenyl group; descriptors had guaranteed good intestinal absorption and a can penetration into the blood-brain barrier. The compound also has a size compatible with DrugLead.


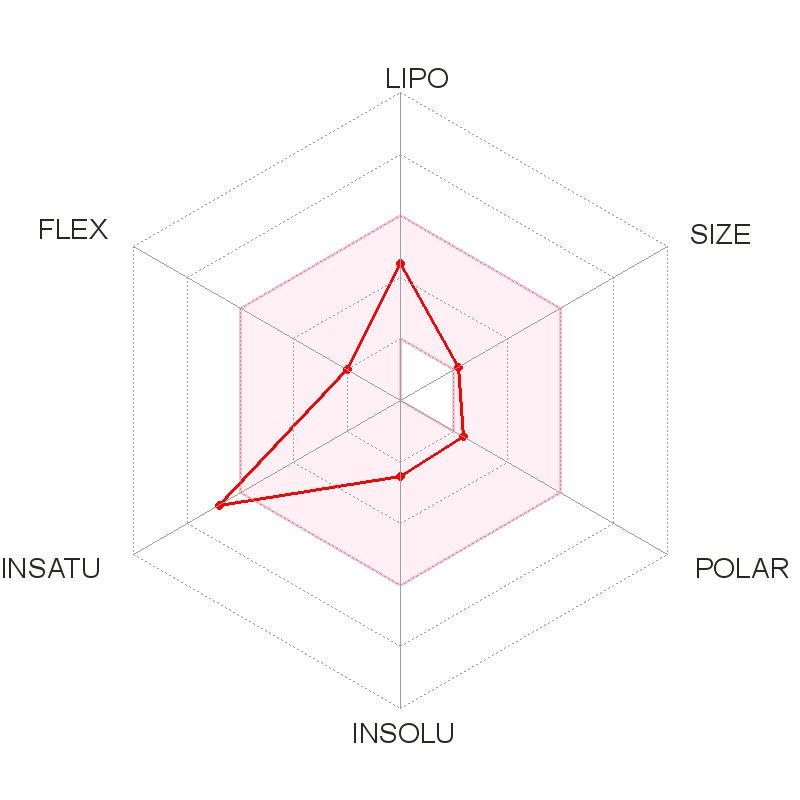

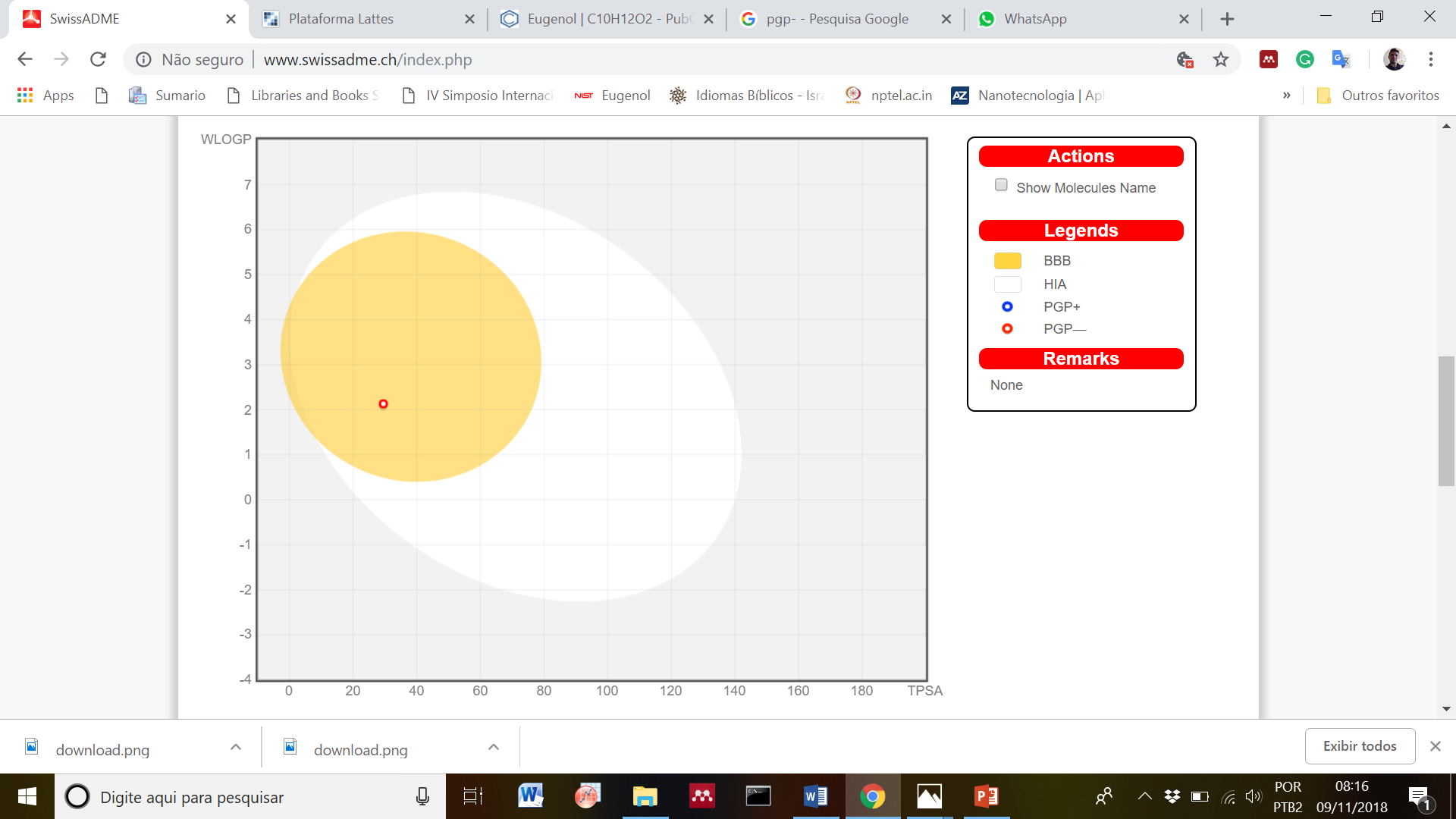

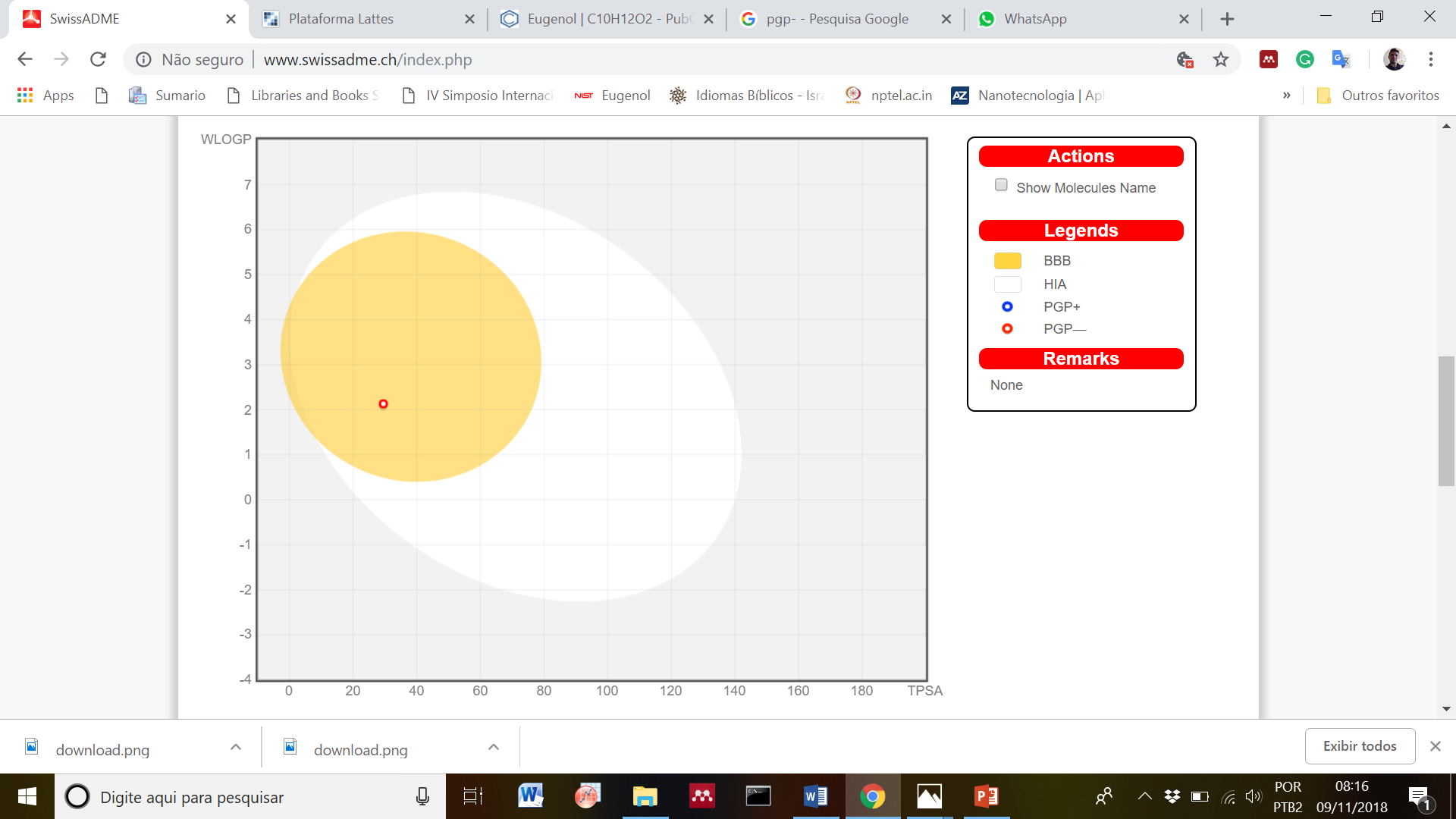


A

B

**Supplement 2.** Molecular Docking in the COX-2 hydrophobic pocket (A) Arachidonic Acid; (B) Diclofenac; (C) Aspirin; (D) Eugenol. Interactions between (E) Arachidonic Acid; (F) Diclofenac; (G) Aspirin; (H) Eugenol and Cox-2 amino acids fragments. All the structures ware generated in Discovery Studio software version 2016 (http://bioviaonline.com/).


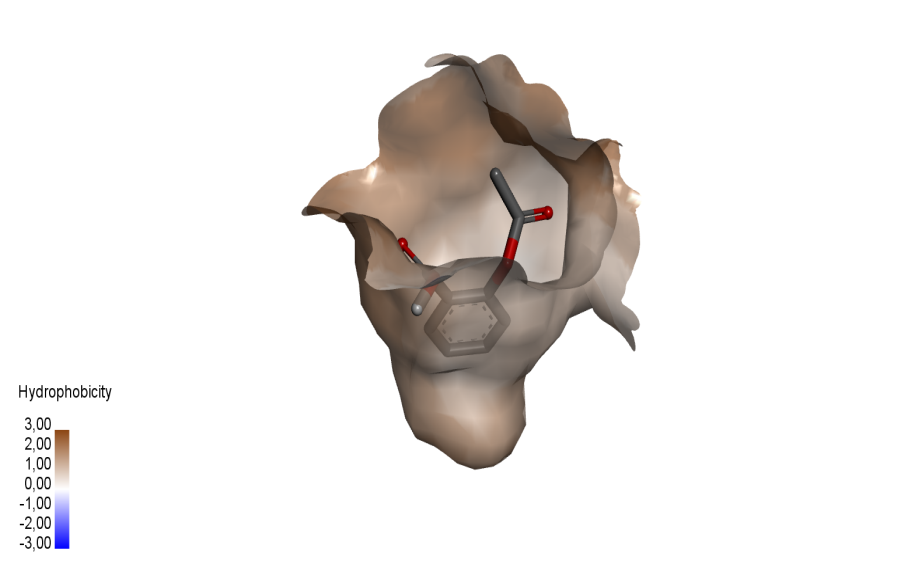

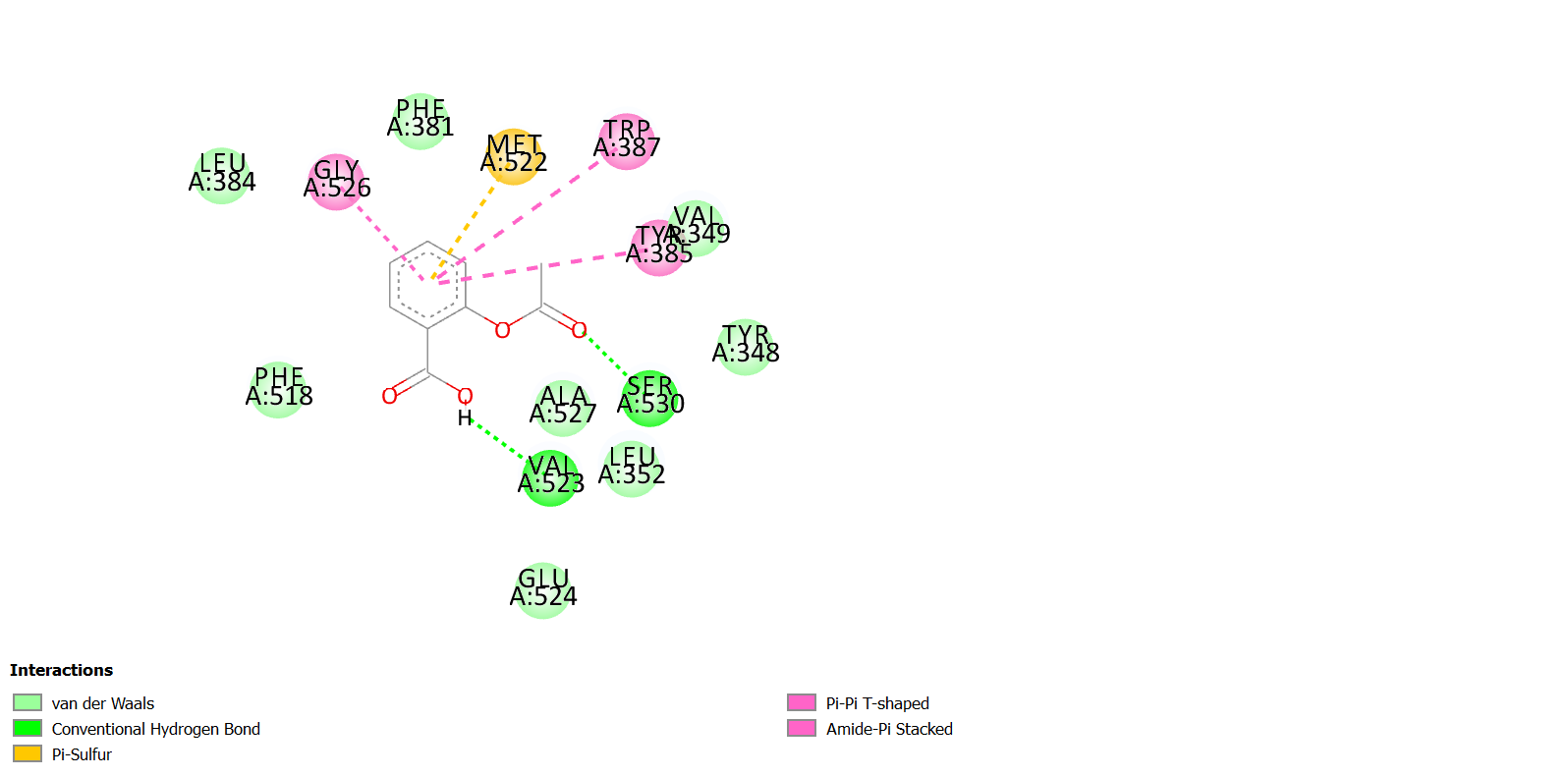

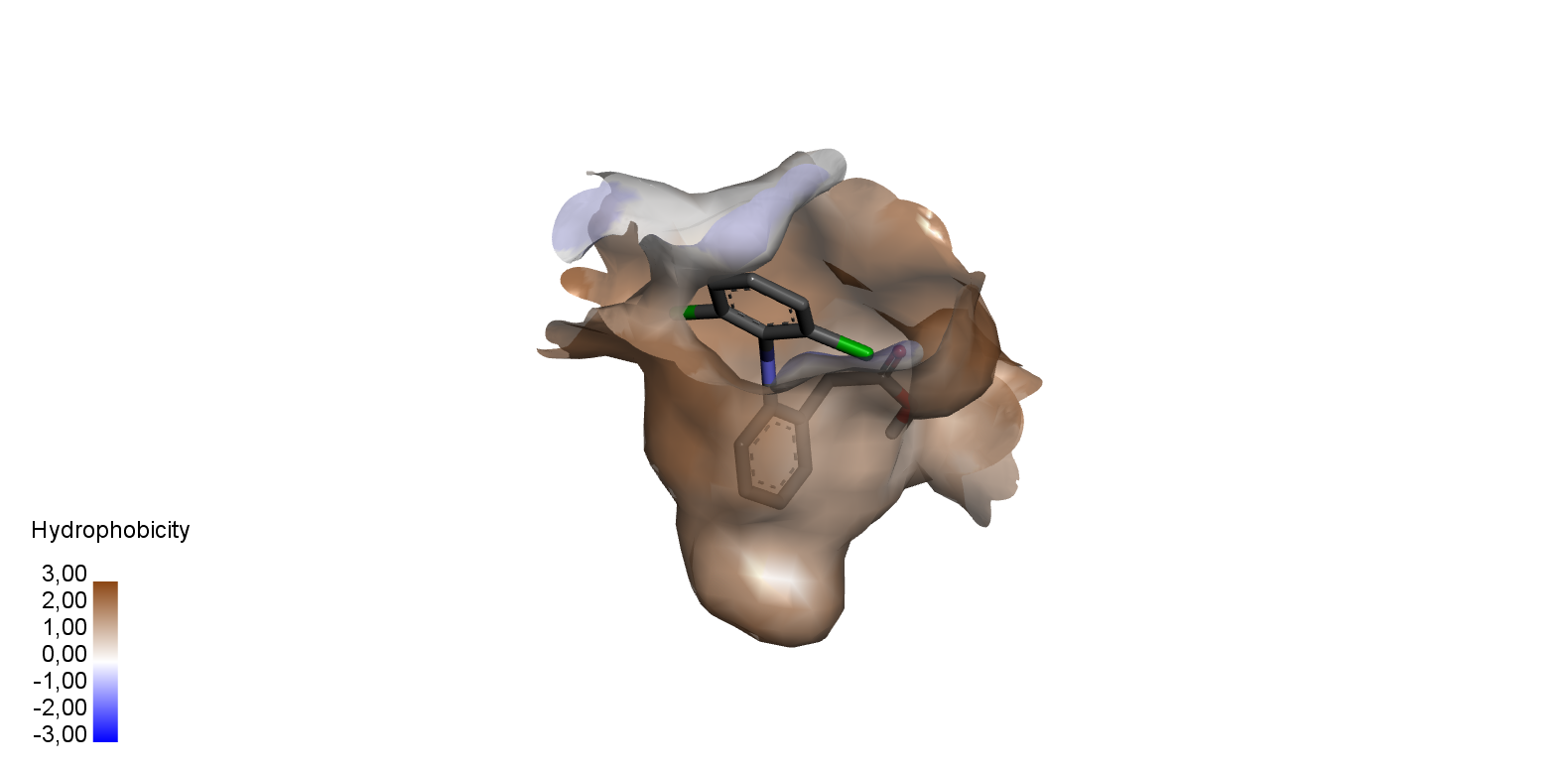

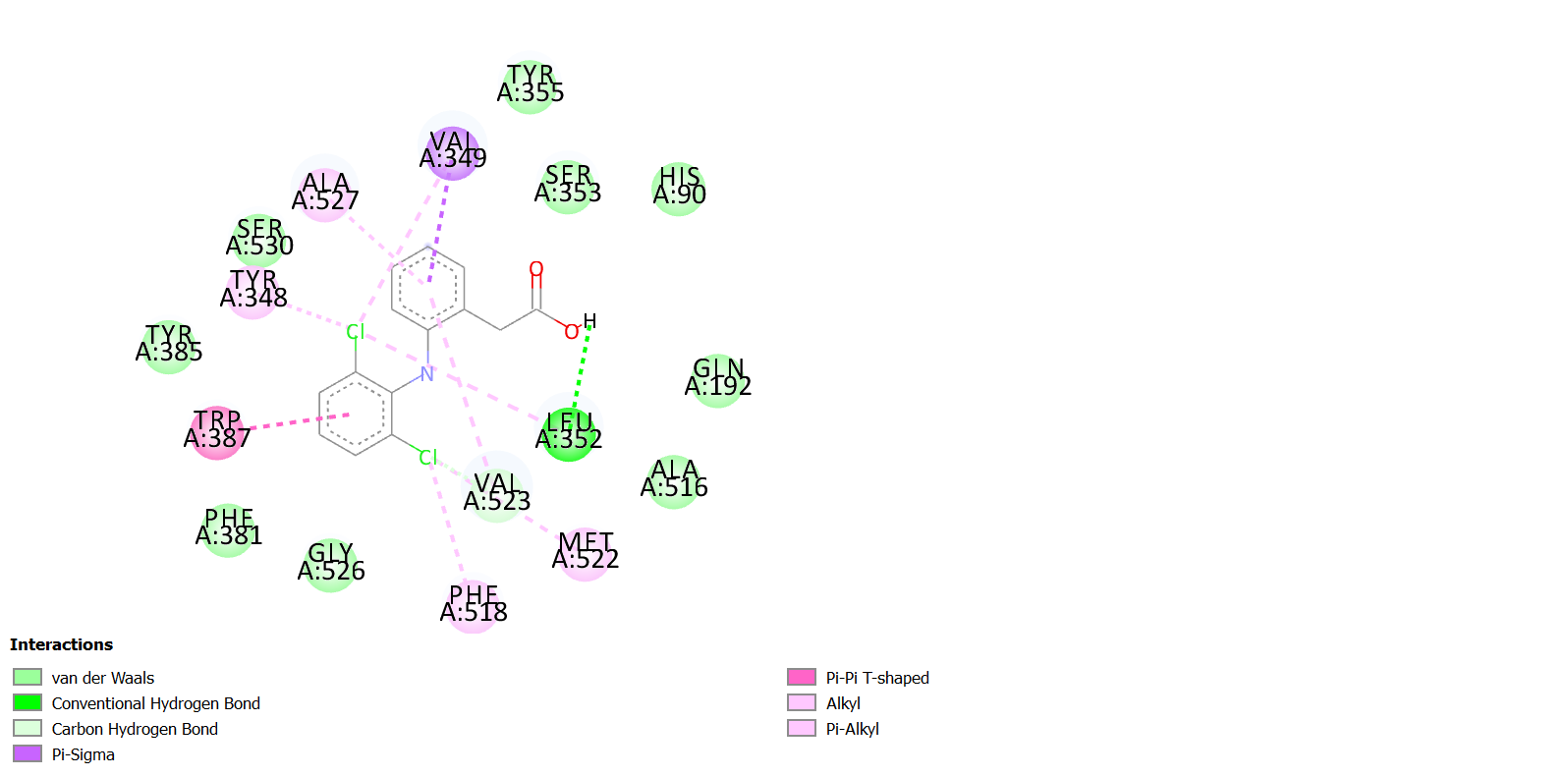

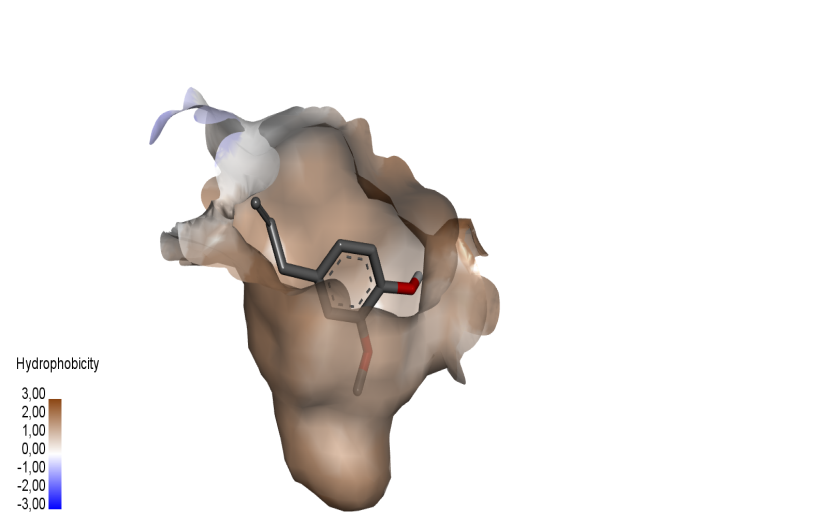

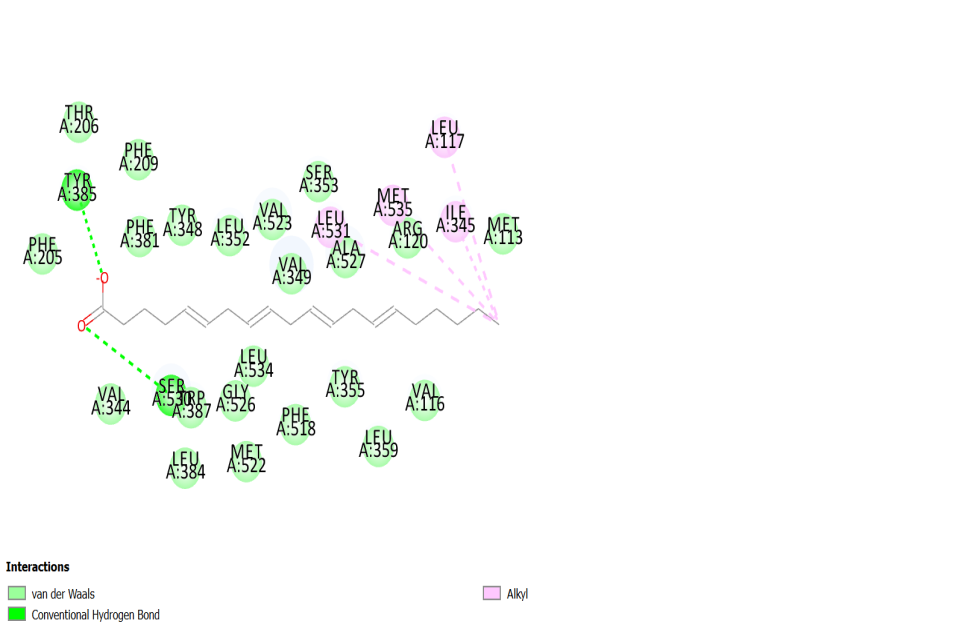

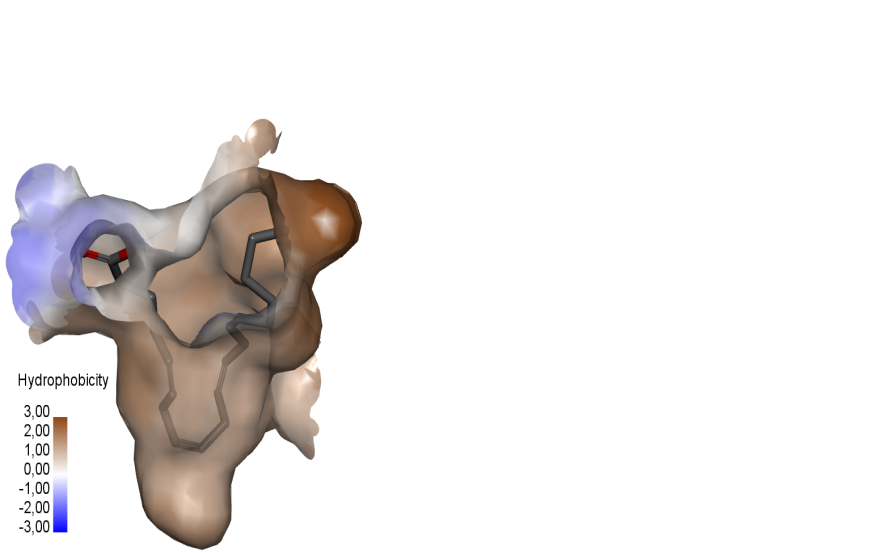


**A**

**C**

**B**

**D**


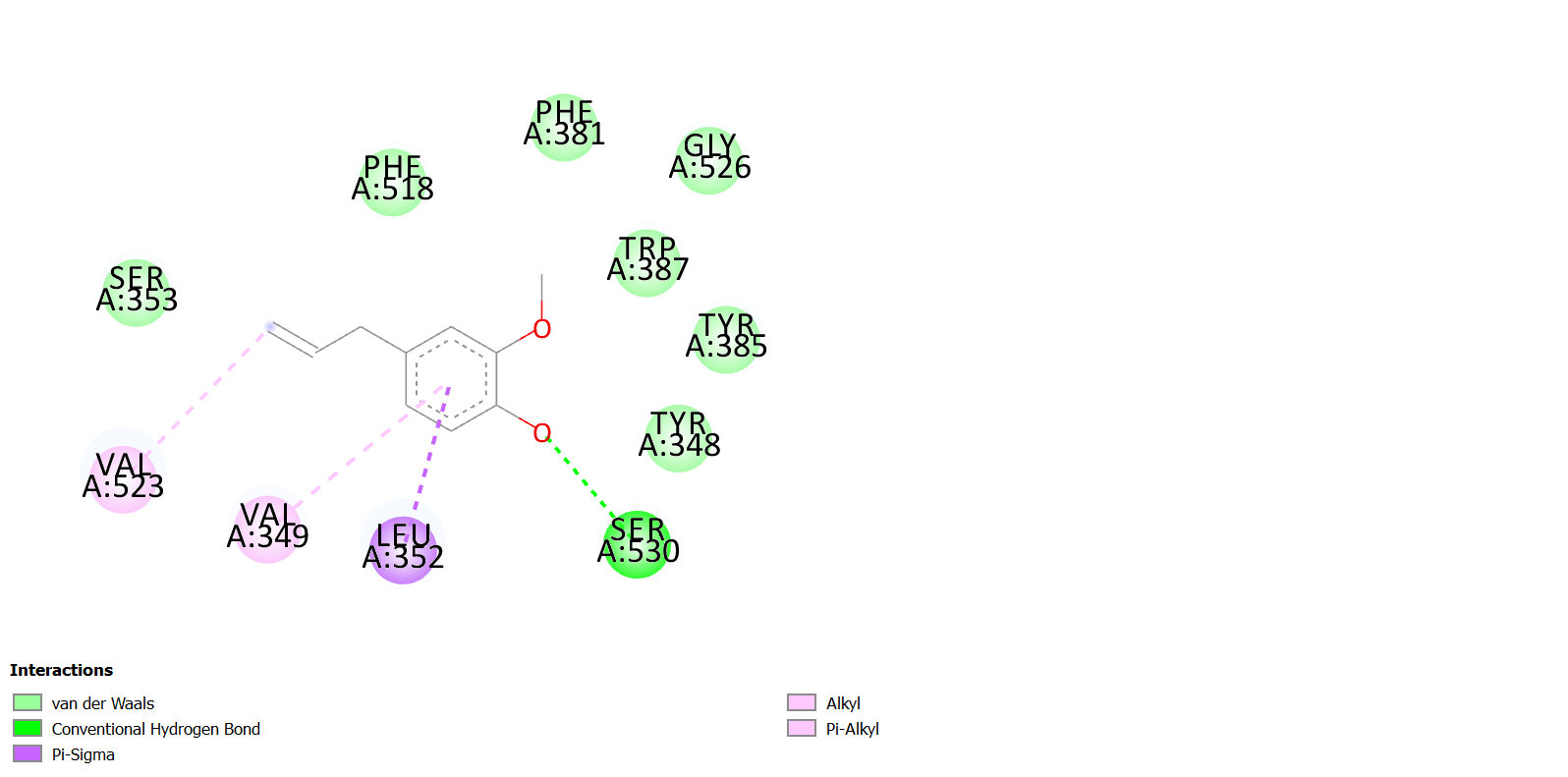


**E**

**F**

**G**

**H**


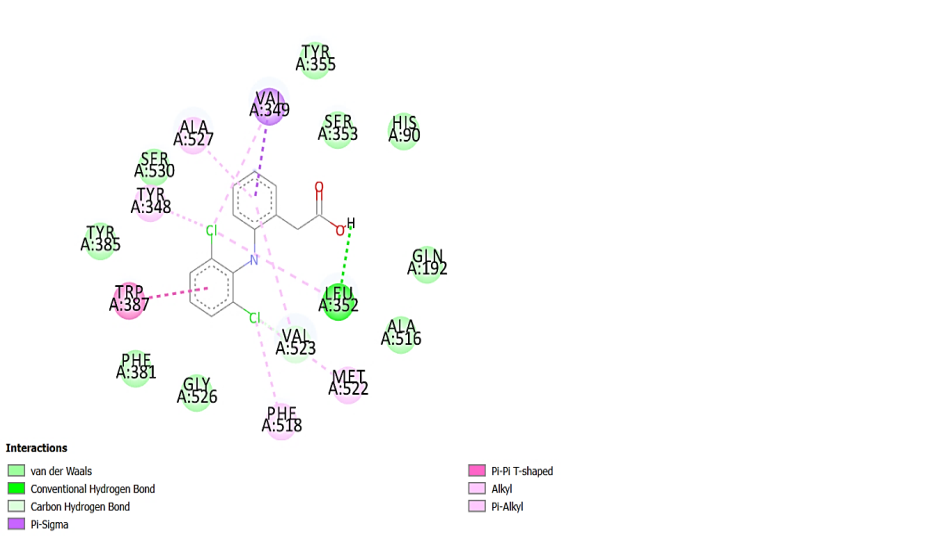

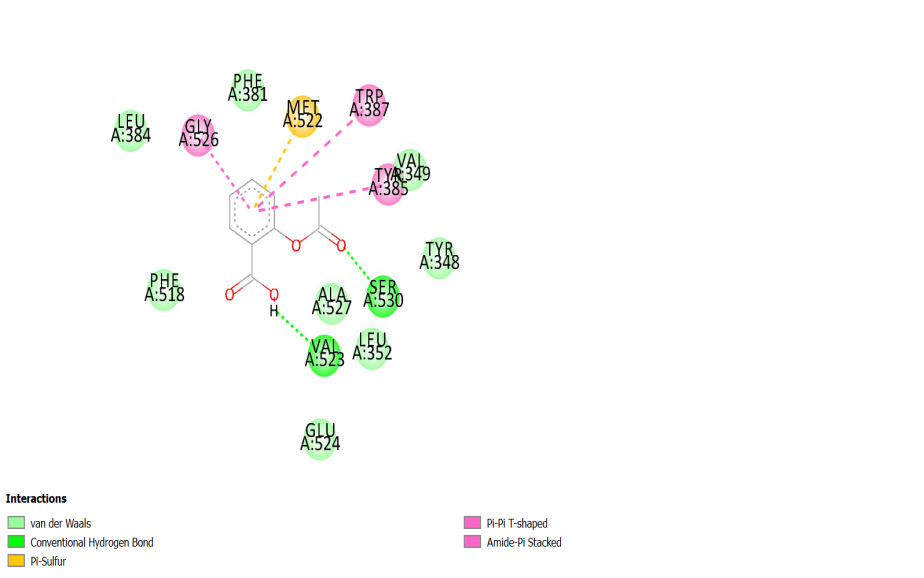

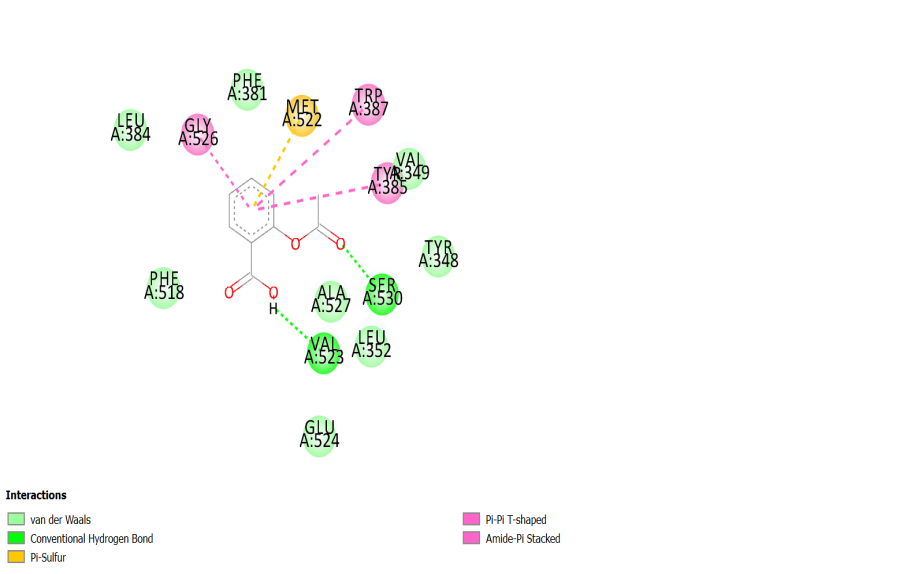


**Supplement 3.** Molecular Docking in the COX-2 peroxidase active pocket (A) Arachidonic Acid; (B) Aspirin; (C) Eugenol. Interactions between (D) Arachidonic Acid; (E) Aspirin; (F) Eugenol and Cox-2 amino acids fragments All the structures ware generated in Discovery Studio software version 2016 (http://bioviaonline.com/).


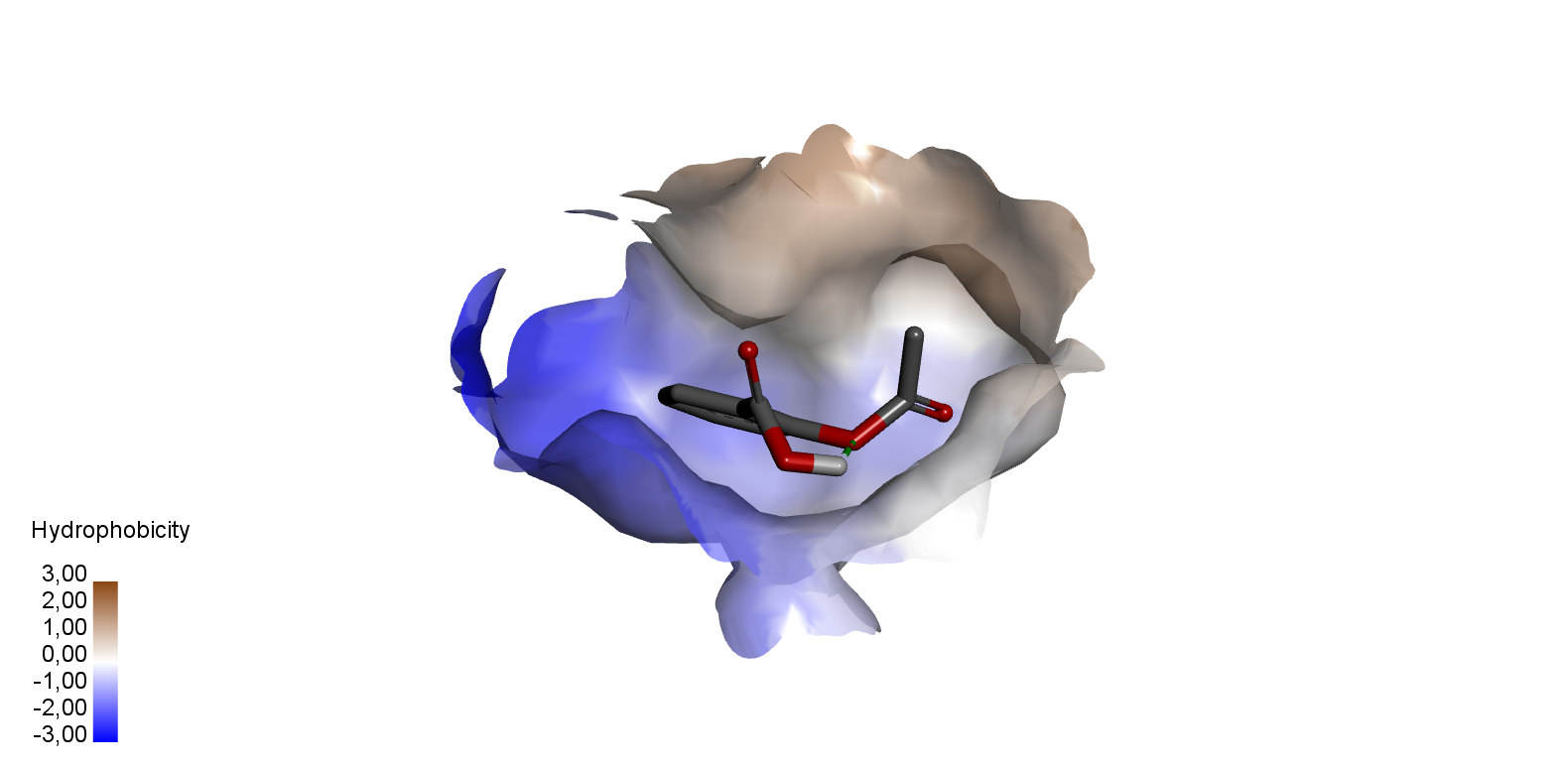

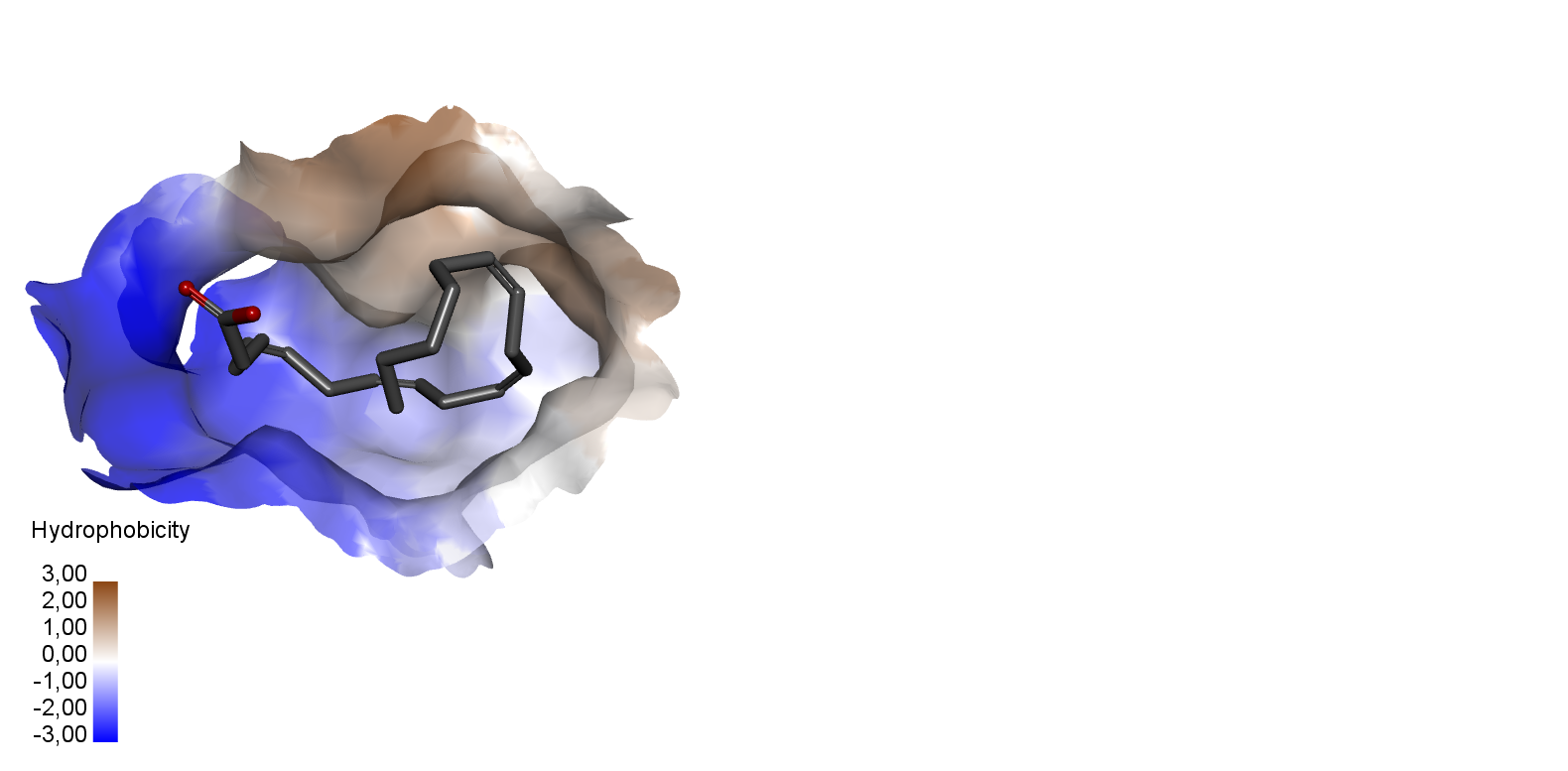

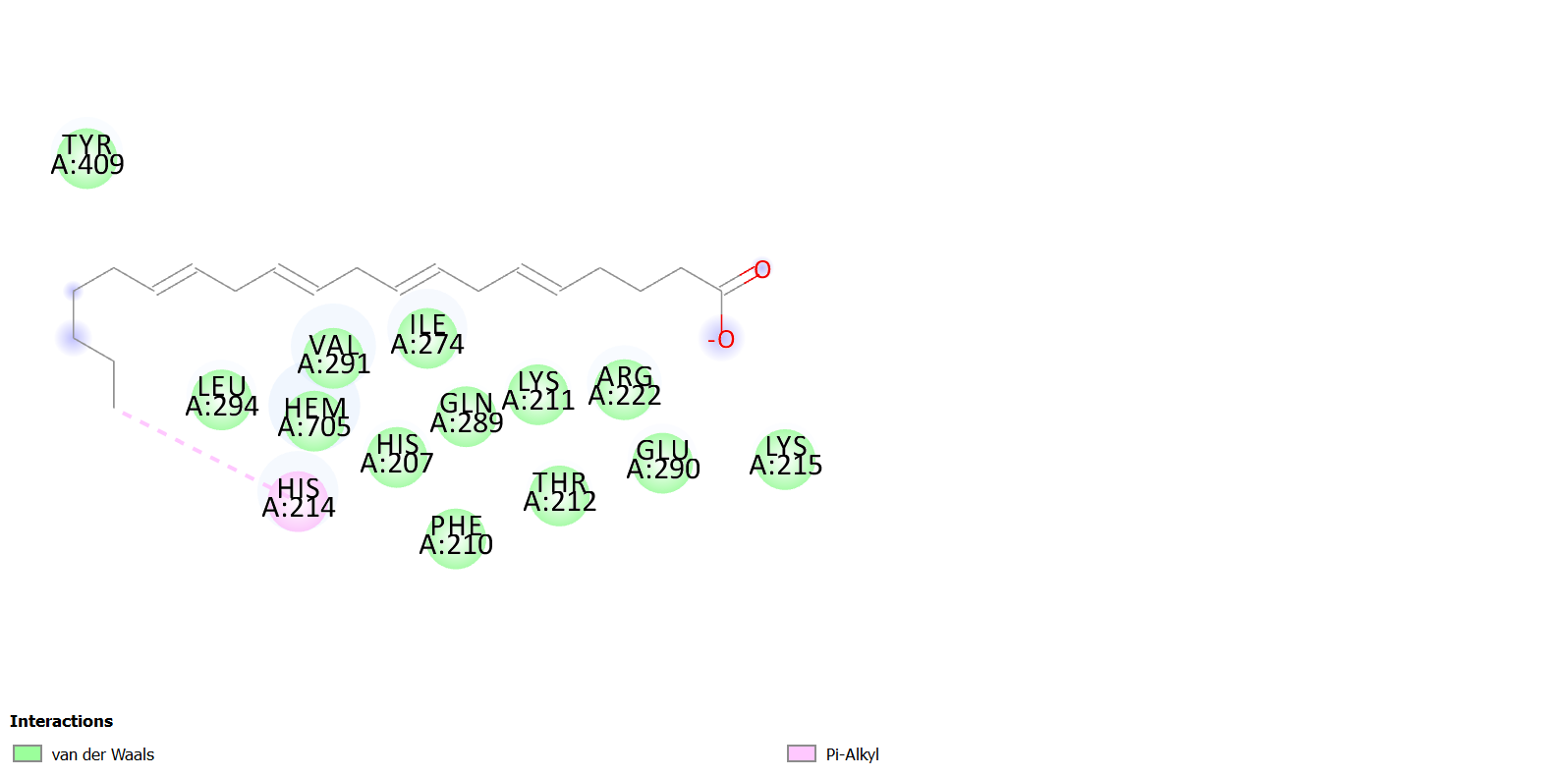


**A**

**D**


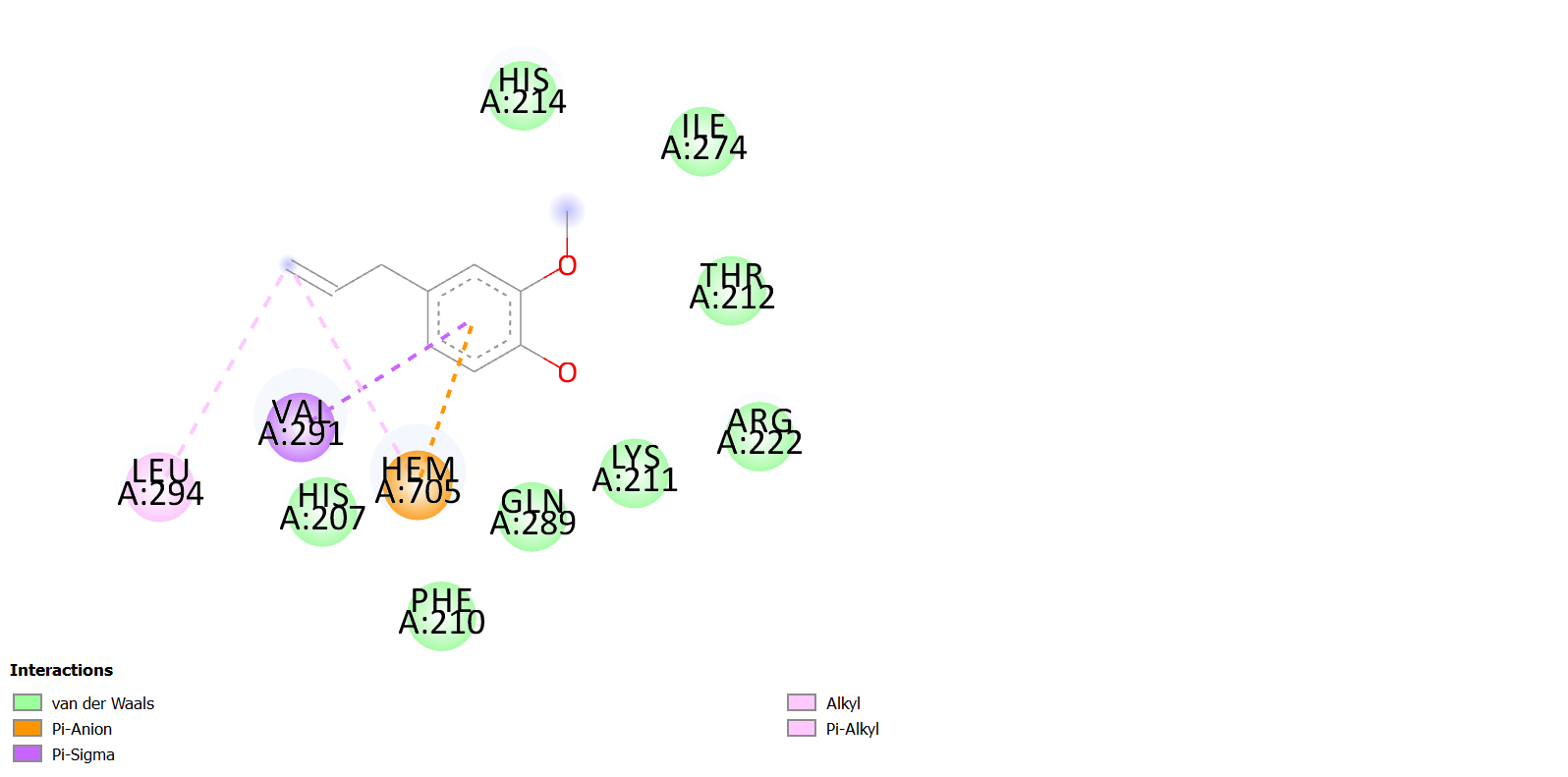

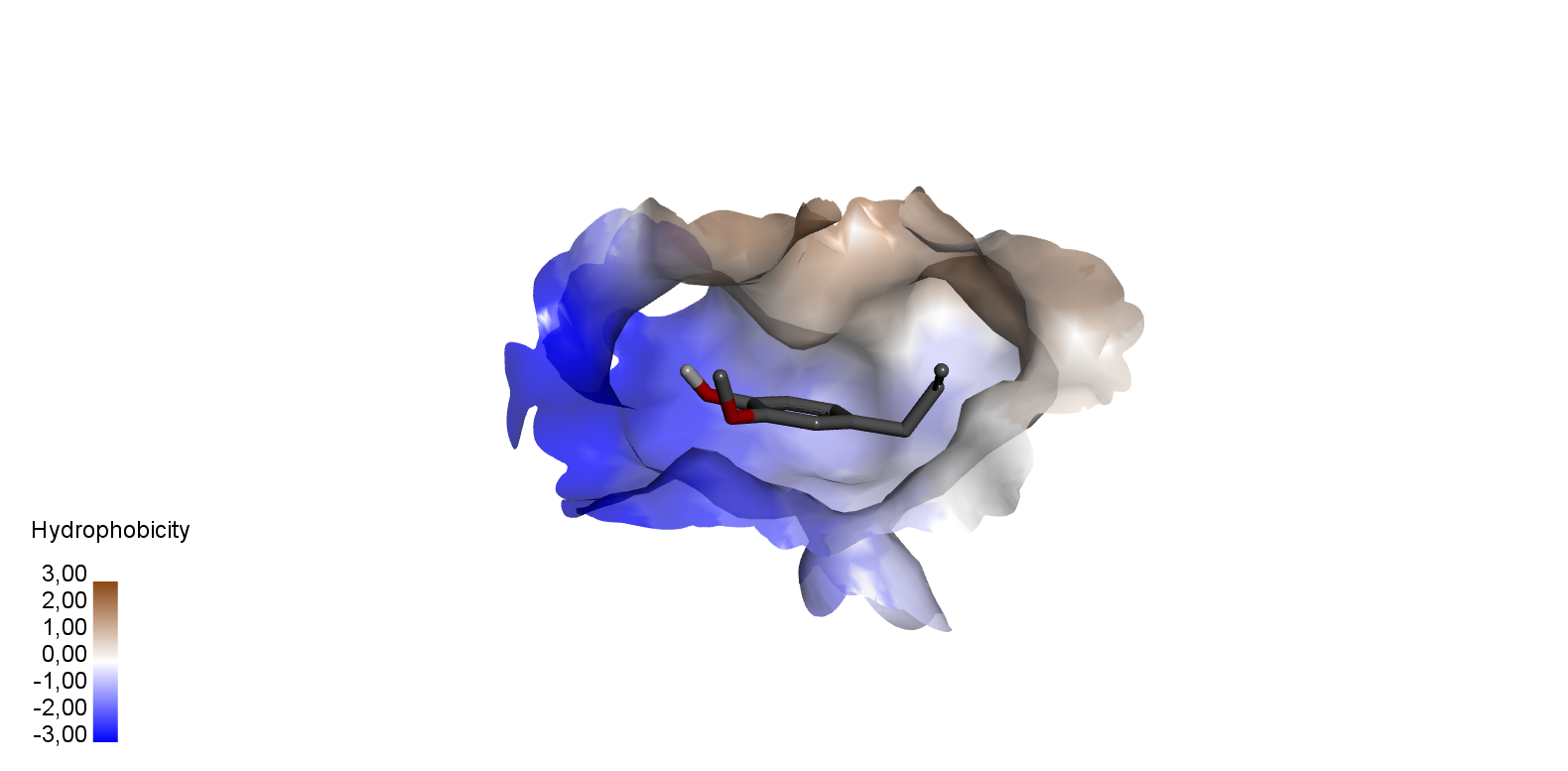


**B**

**E**


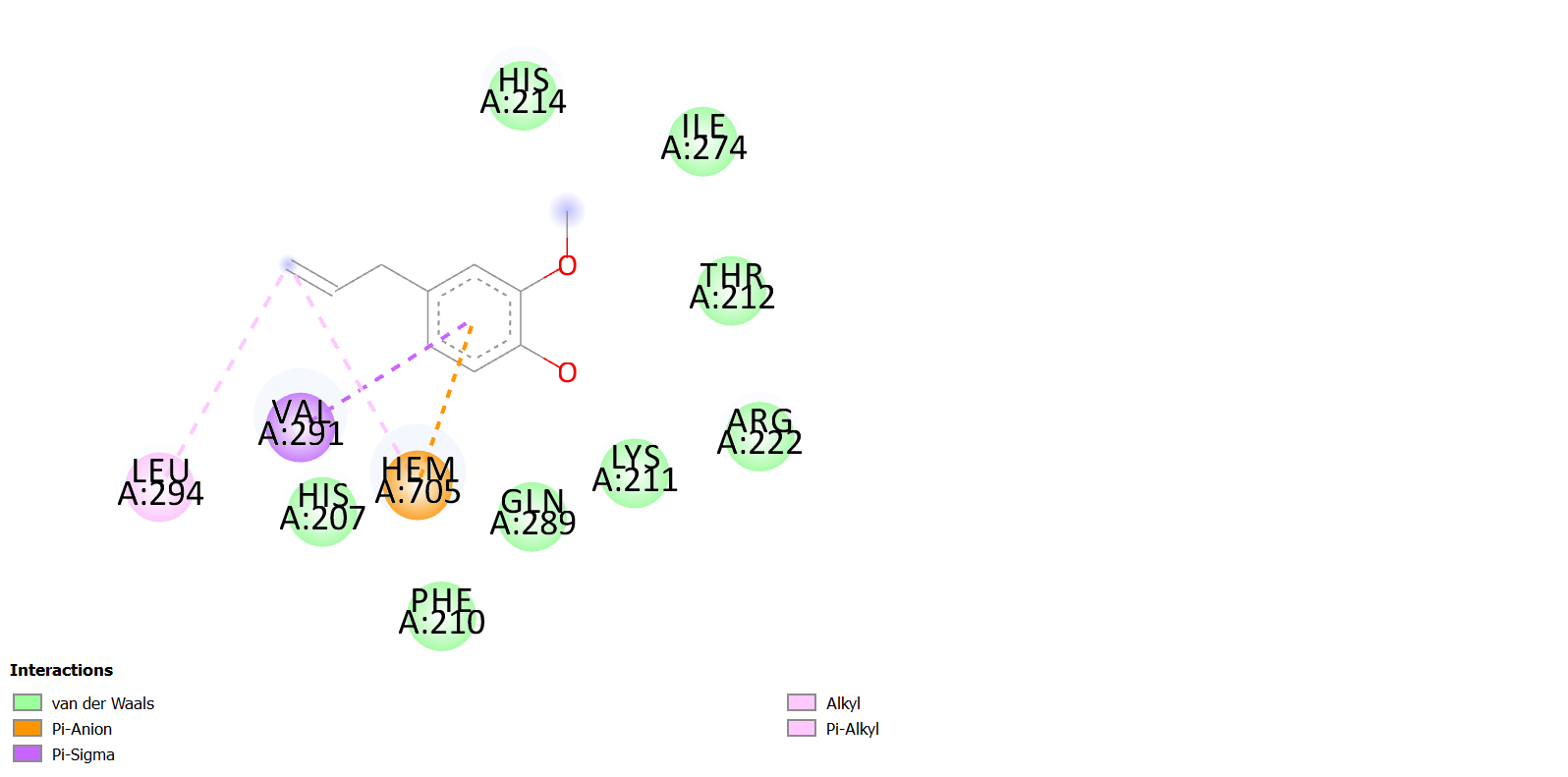


**C**

**F**


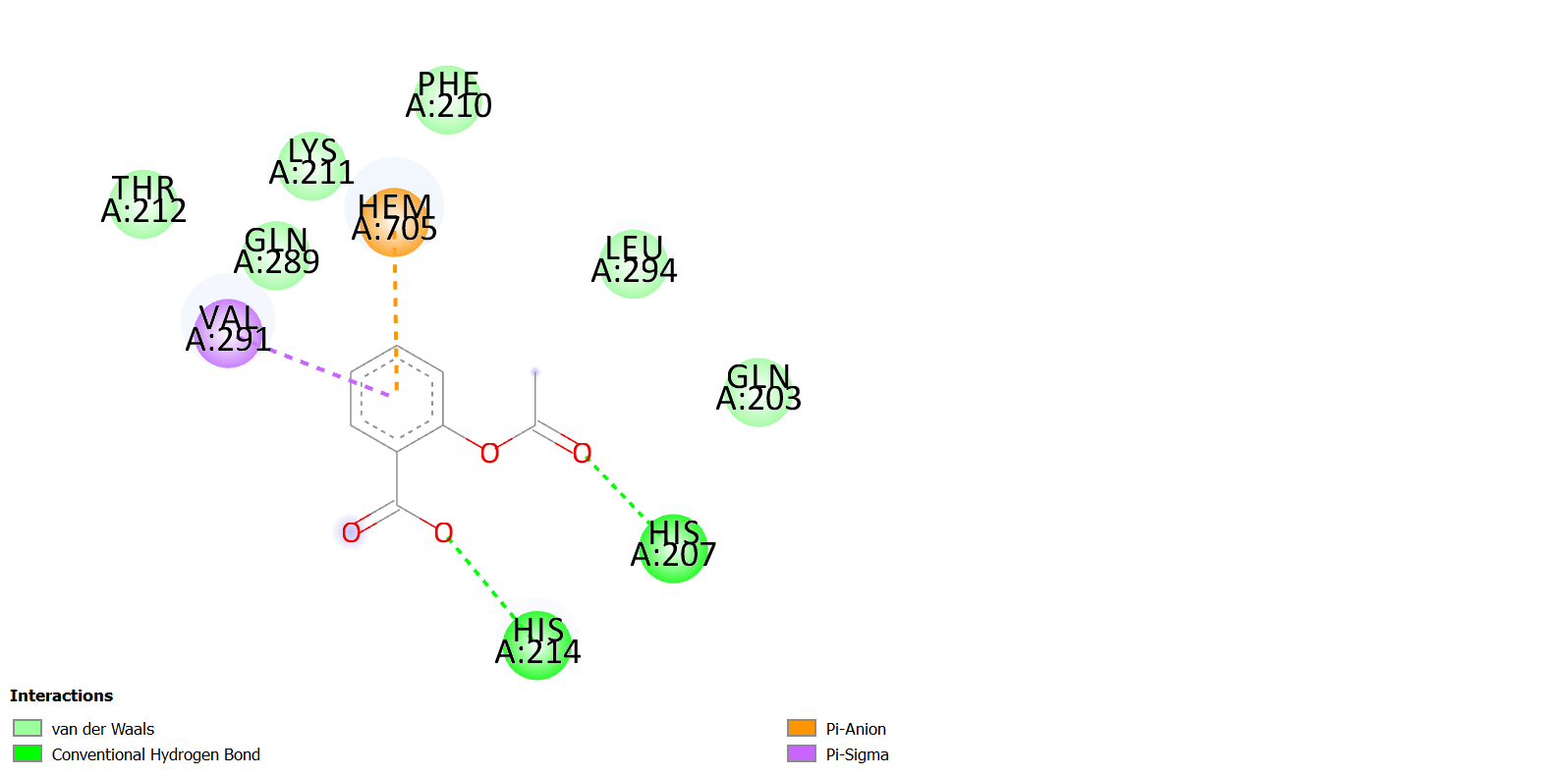


**Supplement 4.** Surface charge density of the eugenol, diclofenac and aspirin molecules, the figure evidence the surface area and respective relative charges calculated by the Gasteiger method, where it observes in red the regions of negative potential and in blue the regions with positive potential. Also noteworthy is the similarity between the structures as to the ease of hydroxyl deprotonation. The surface charge density maps were generated using IQMol software, version 2.11.1 (http://iqmol.org/).


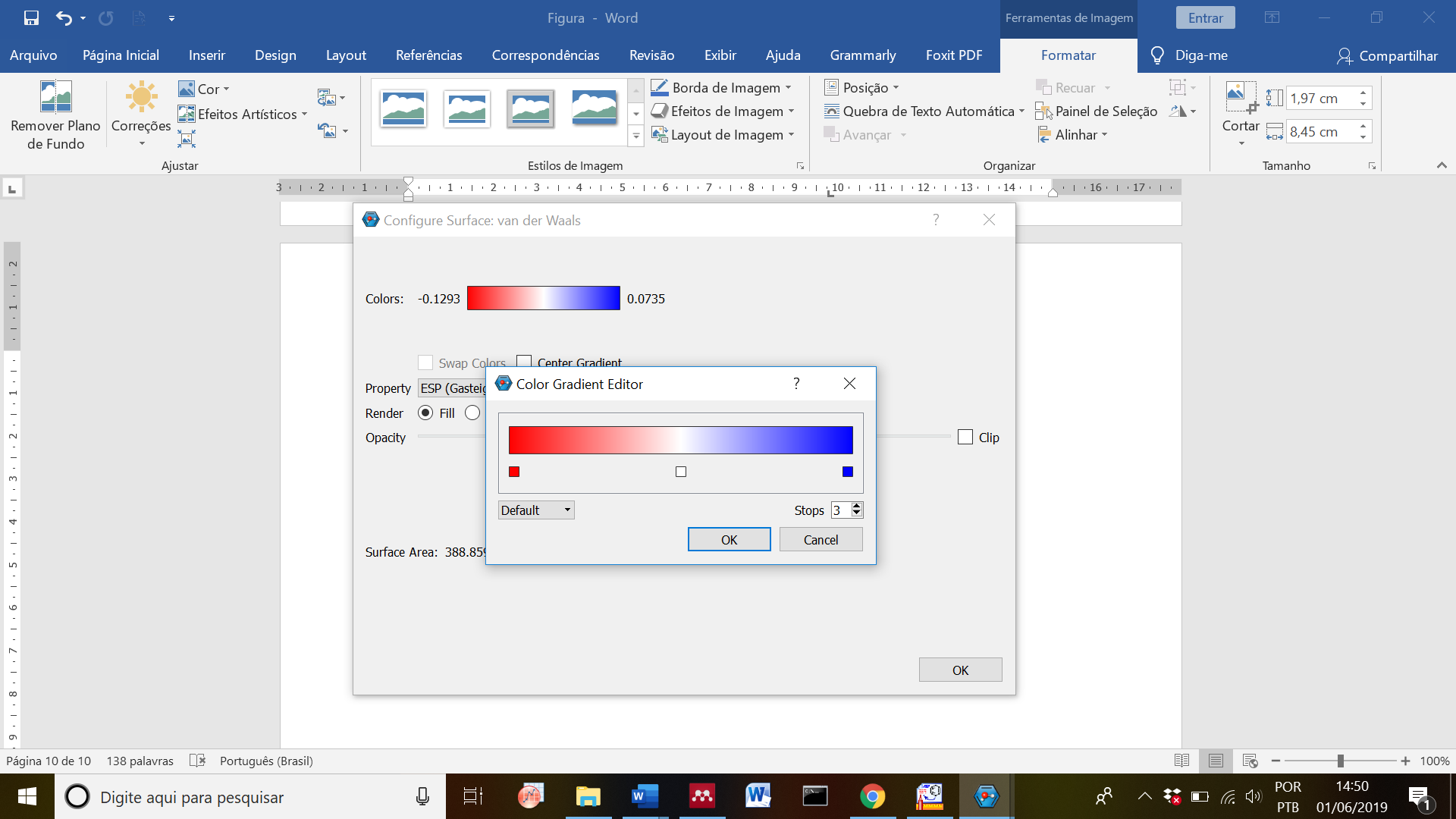


-0,1450

8,8086


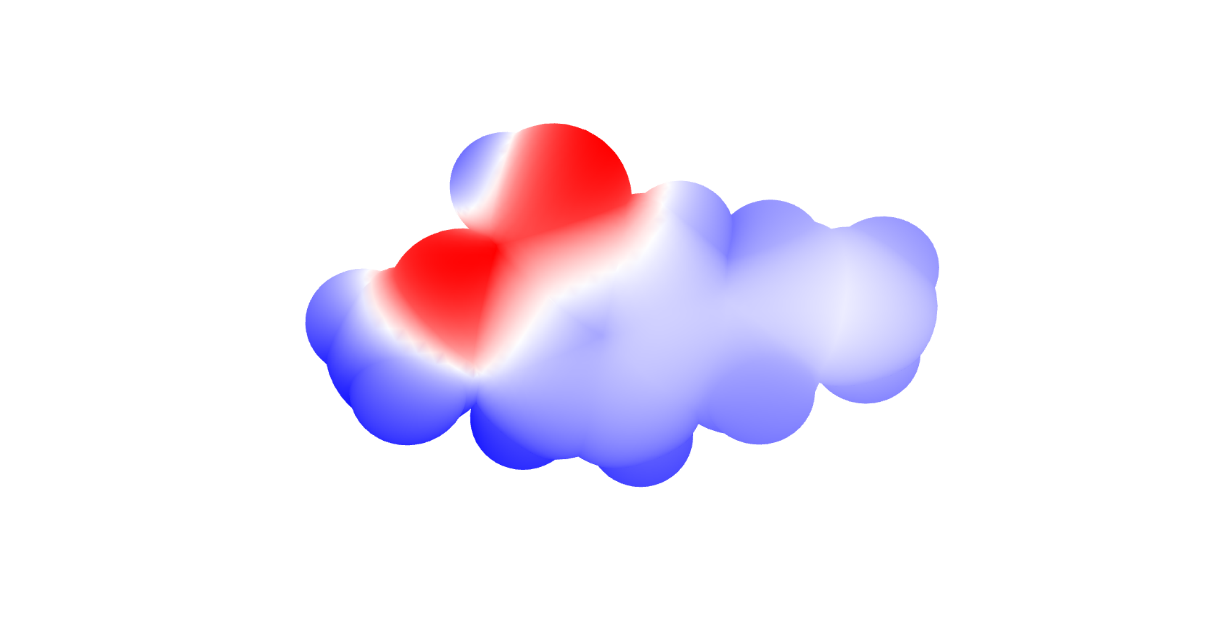


Eugenol


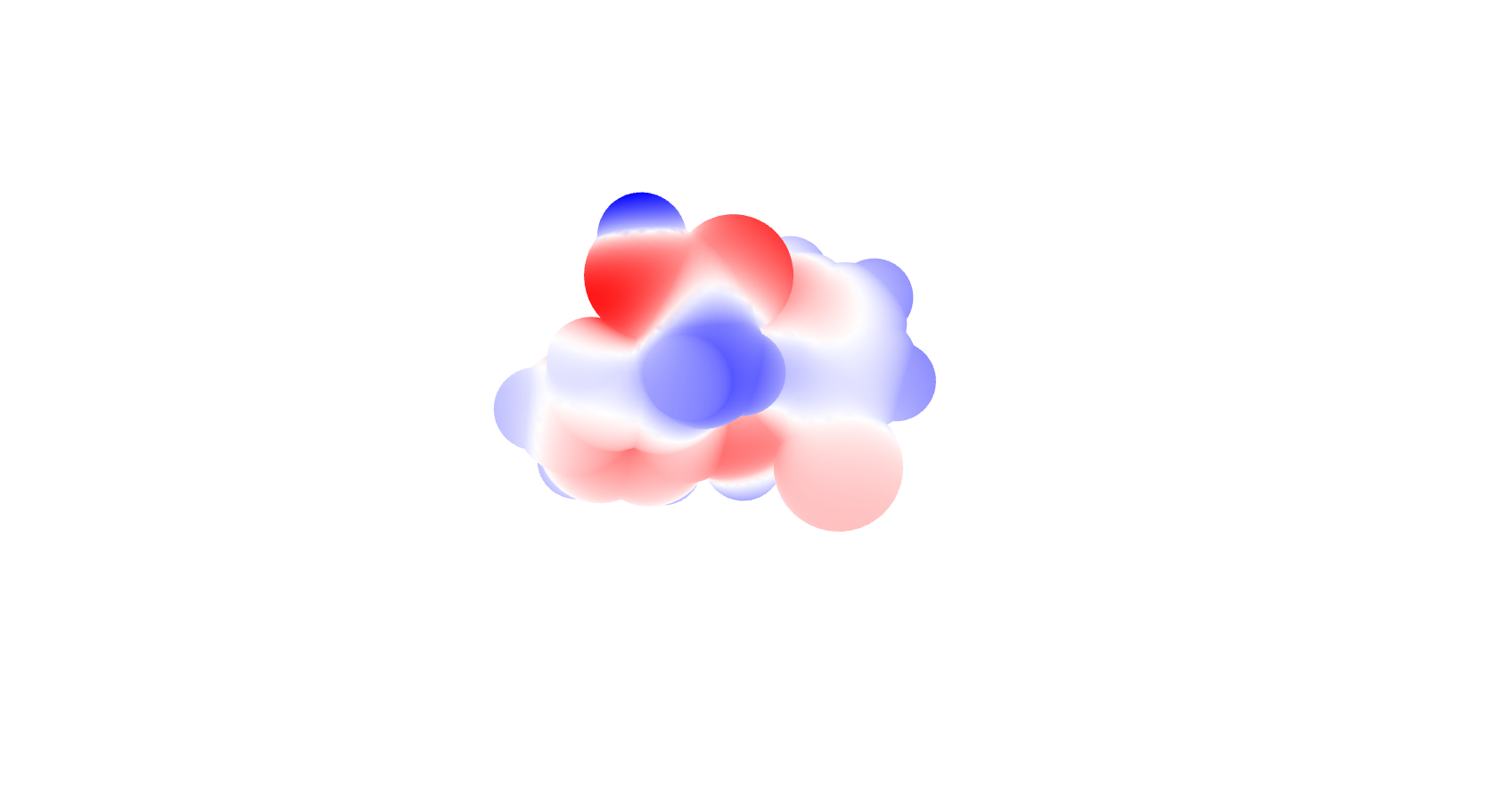


Diclofenac


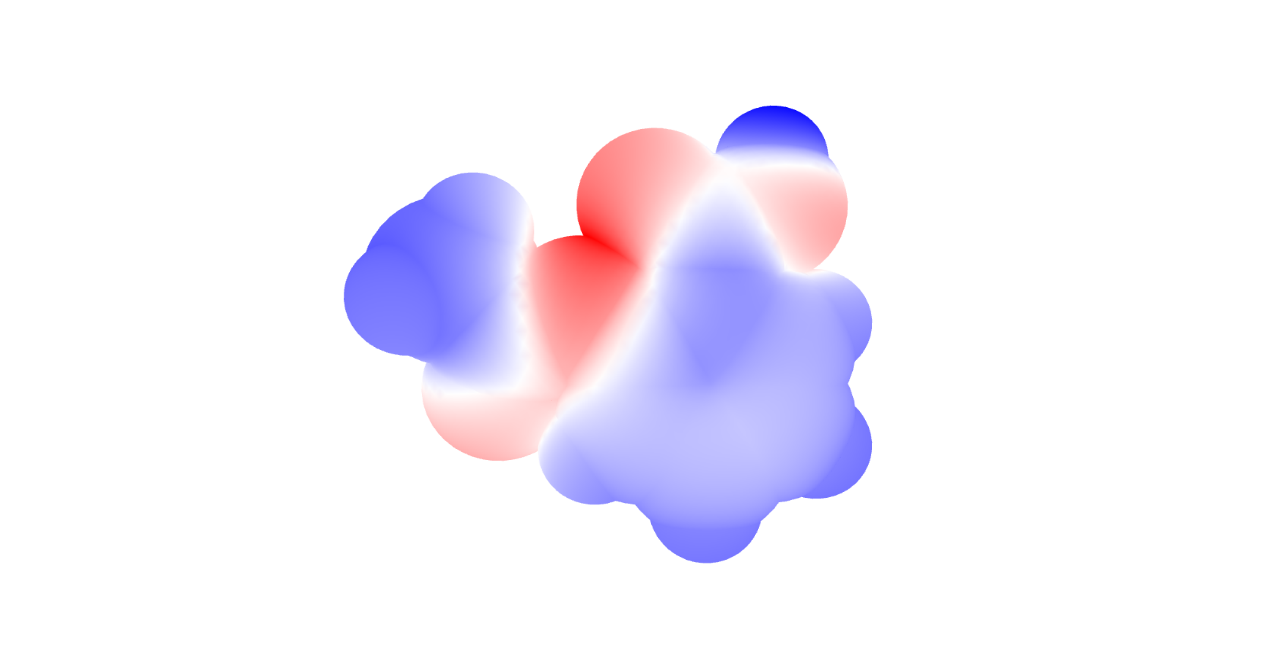


Aspirin

**Supplement 5.** Molecular Docking of eugenol in coordinated to the nonheme catalytic Fe^3+^ His 372 5-LOX active pocket. The figure shows (A) Arachidonic Acid; (B) Eugenol; and Interactions between (C) Arachidonic Acid; (D) Eugenol and Cox-2 amino acids fragments of the active pocket. All the structures ware generated in Discovery Studio software version 2016 (http://bioviaonline.com/).


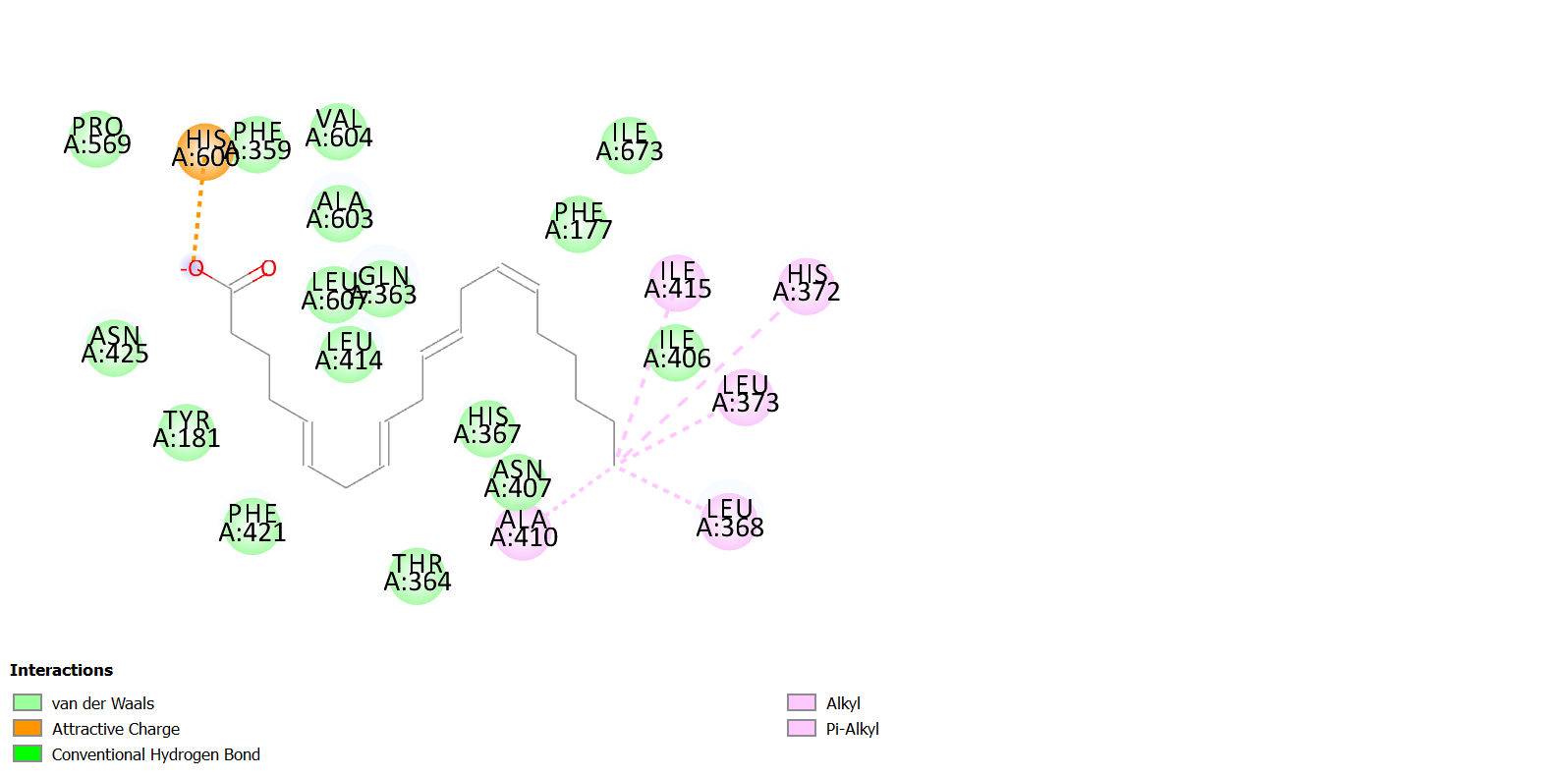

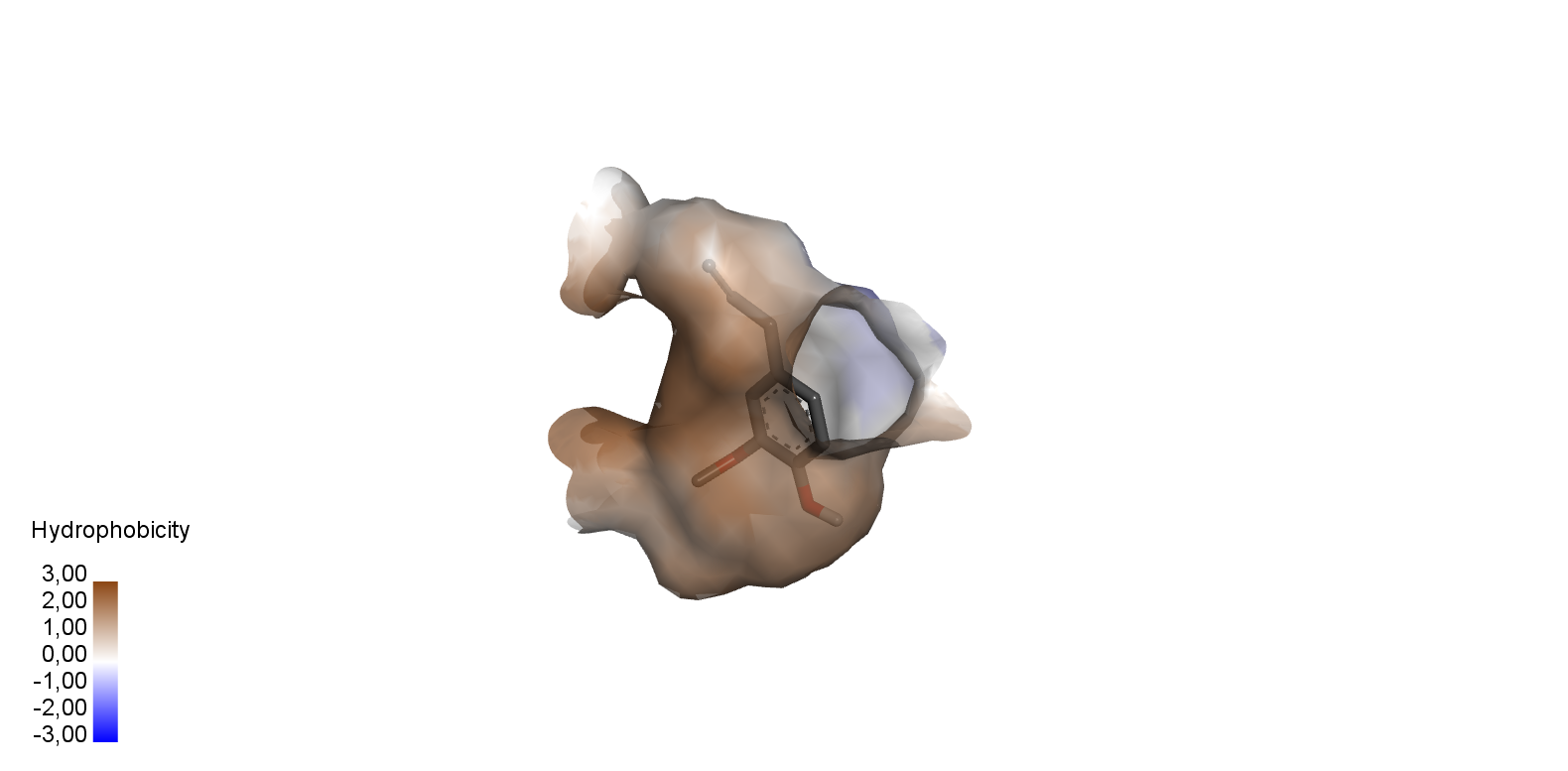

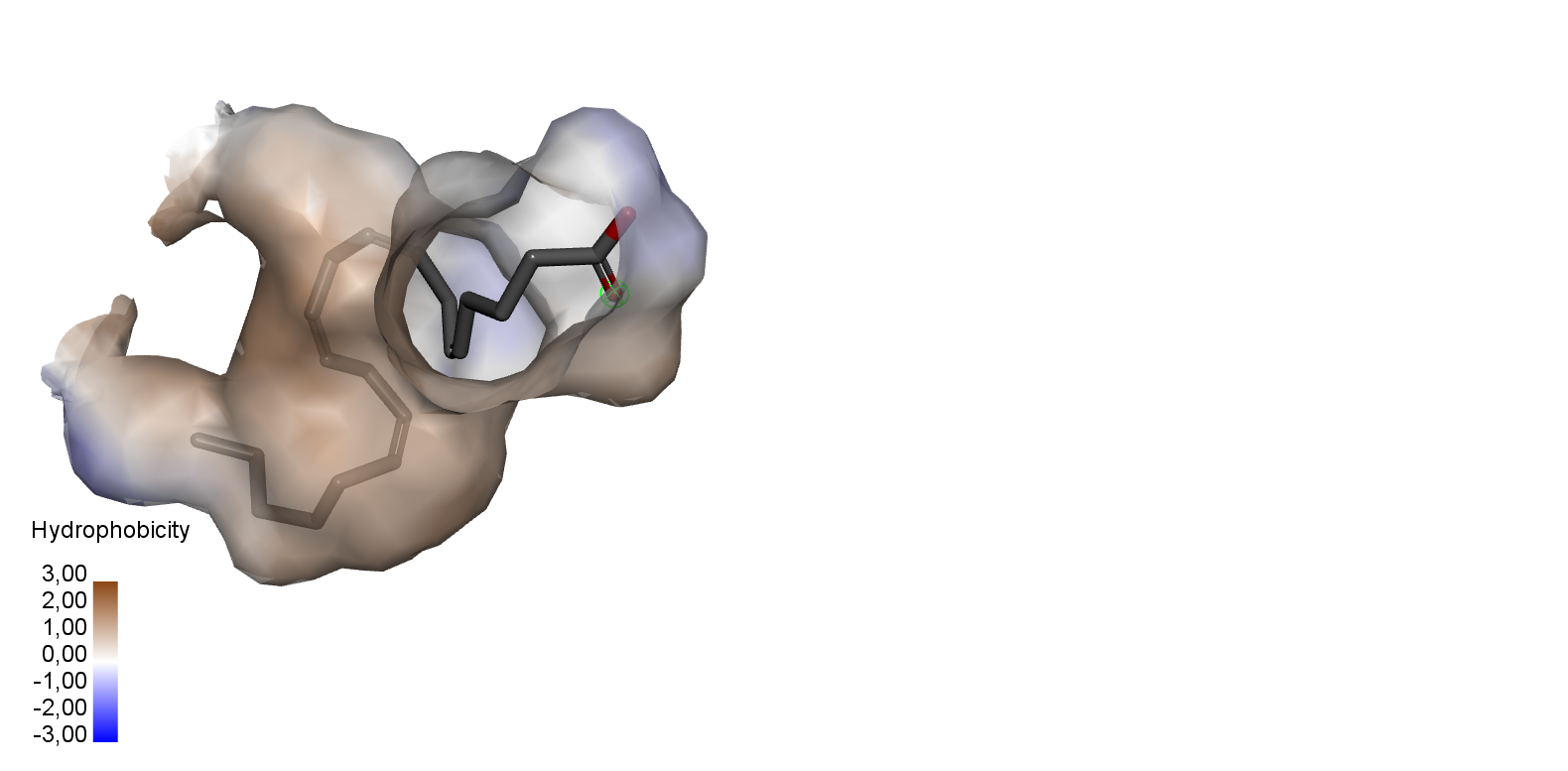


**A**

**C**

**B**

**D**


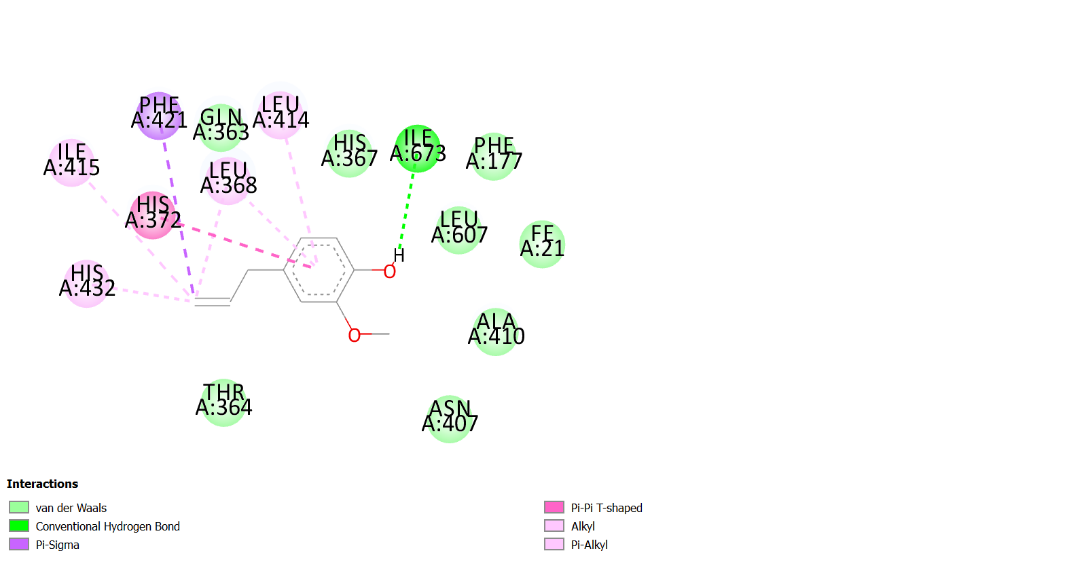

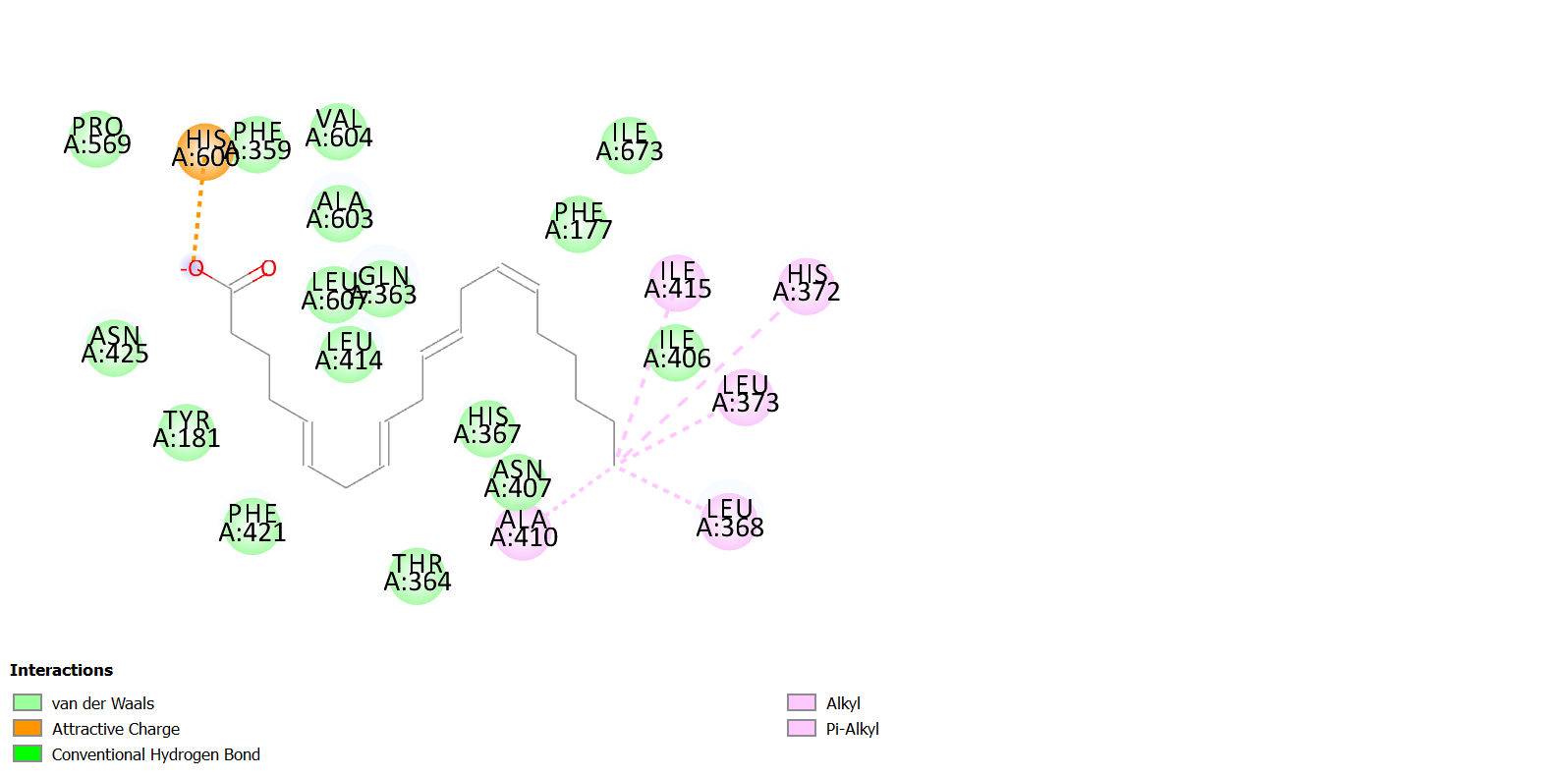

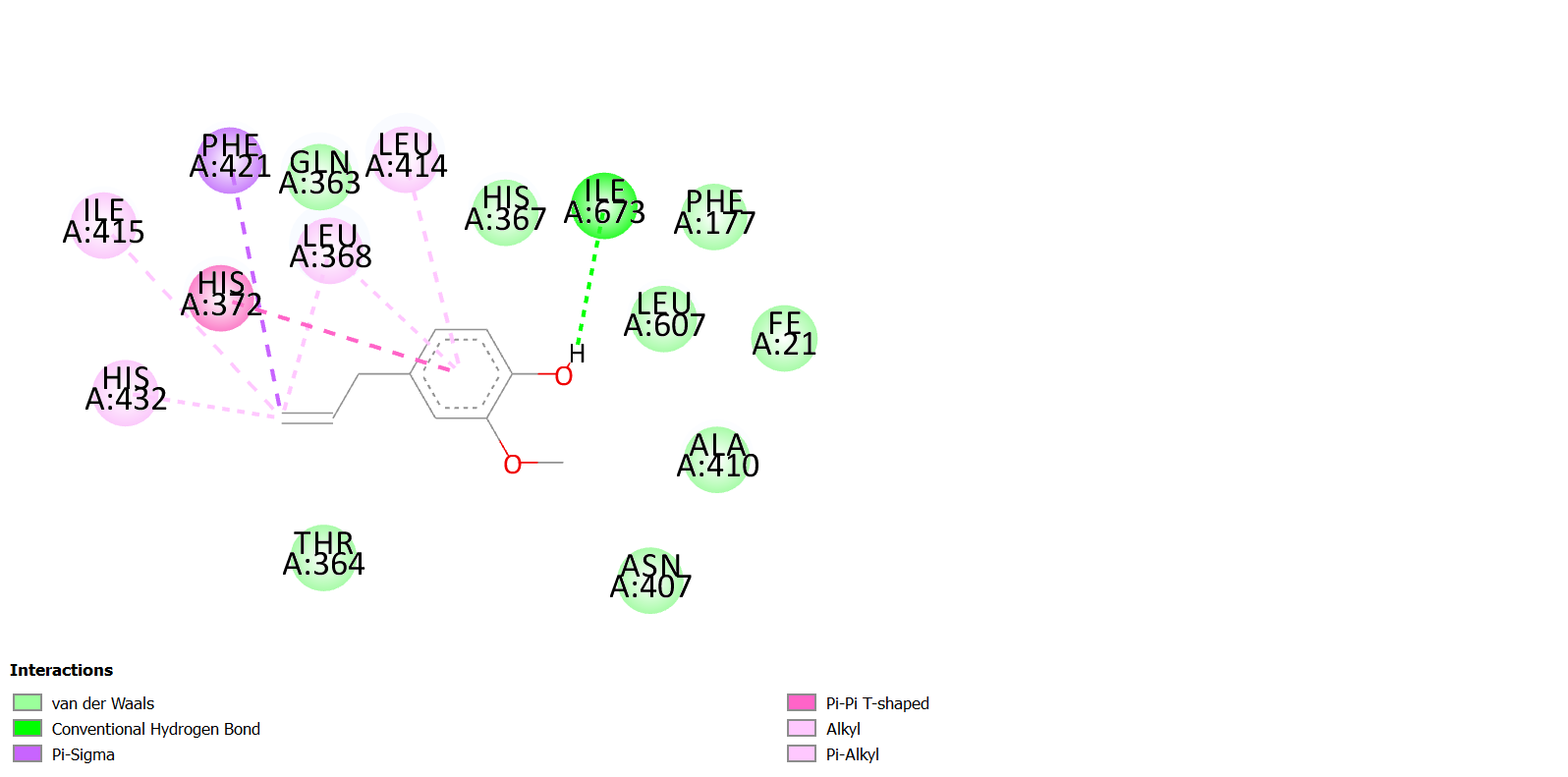


**D**

**Supplement 6.** Molecular Docking of eugenol in additional 5-LOX active pocket. The figure shows (A) Arachidonic Acid; (B) Eugenol; and Interactions between (C) Arachidonic Acid; (D) Eugenol and Cox-2 amino acids fragments. All the structures ware generated in Discovery Studio software version 2016 (http://bioviaonline.com/).


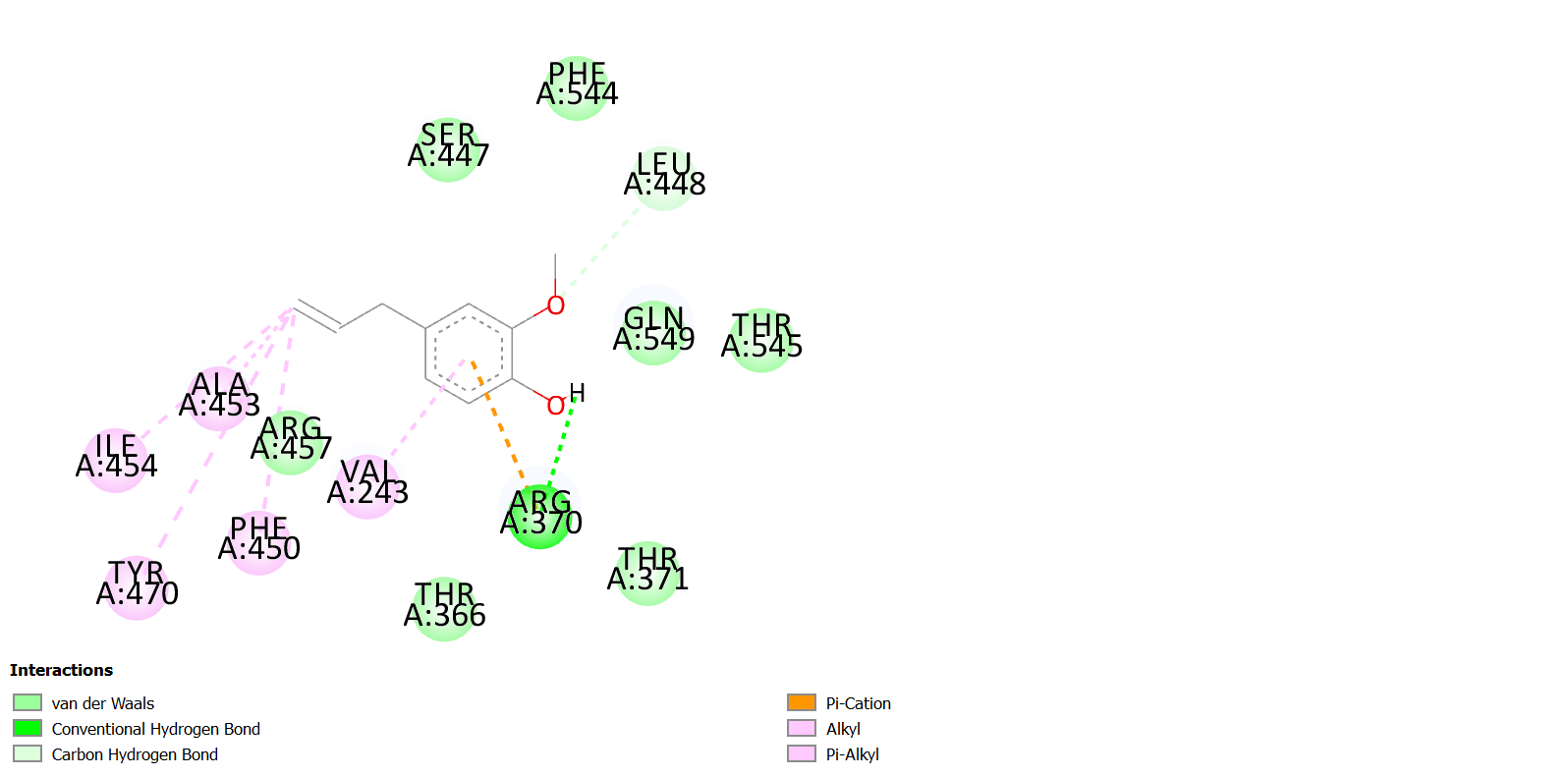

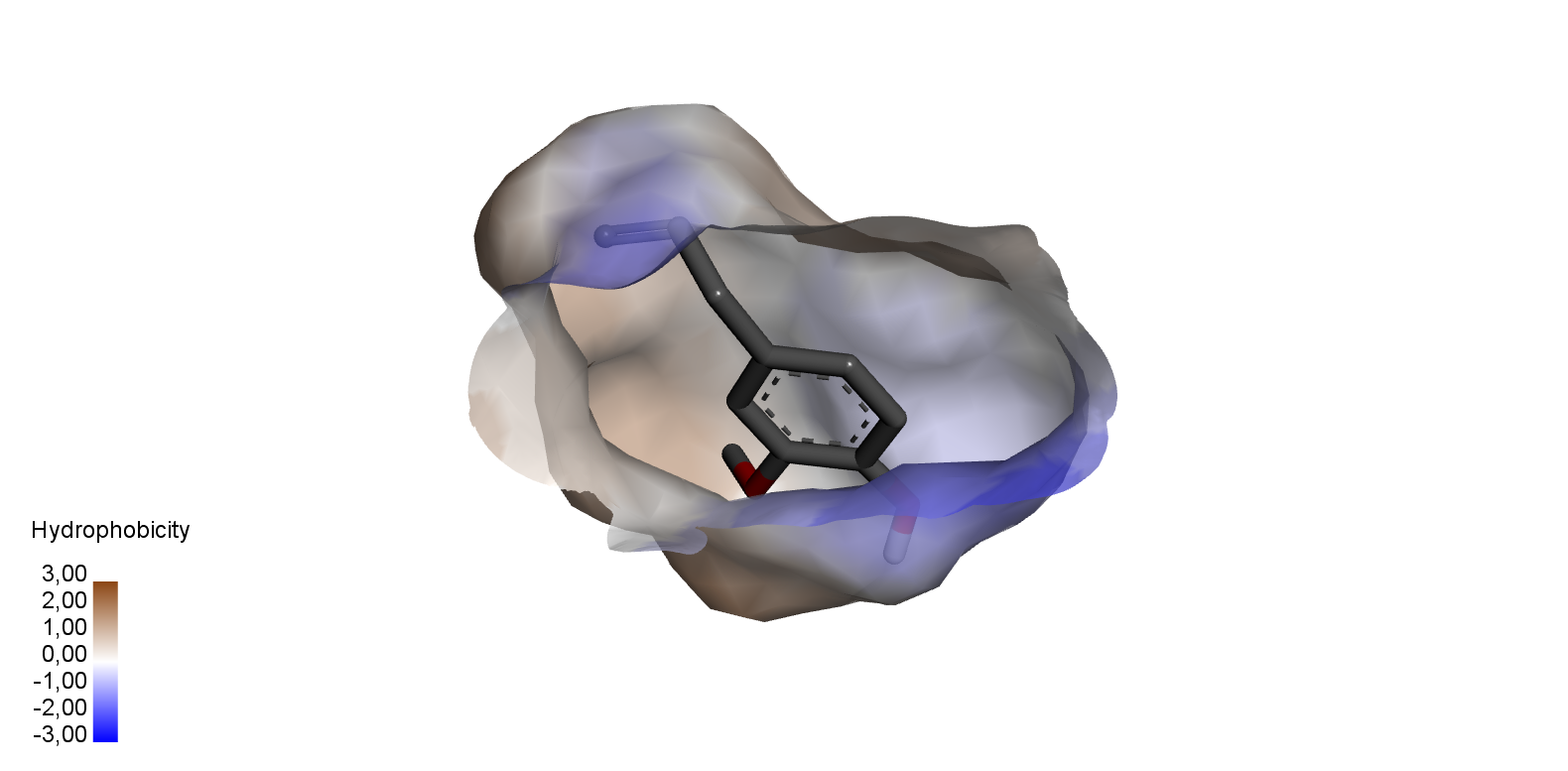

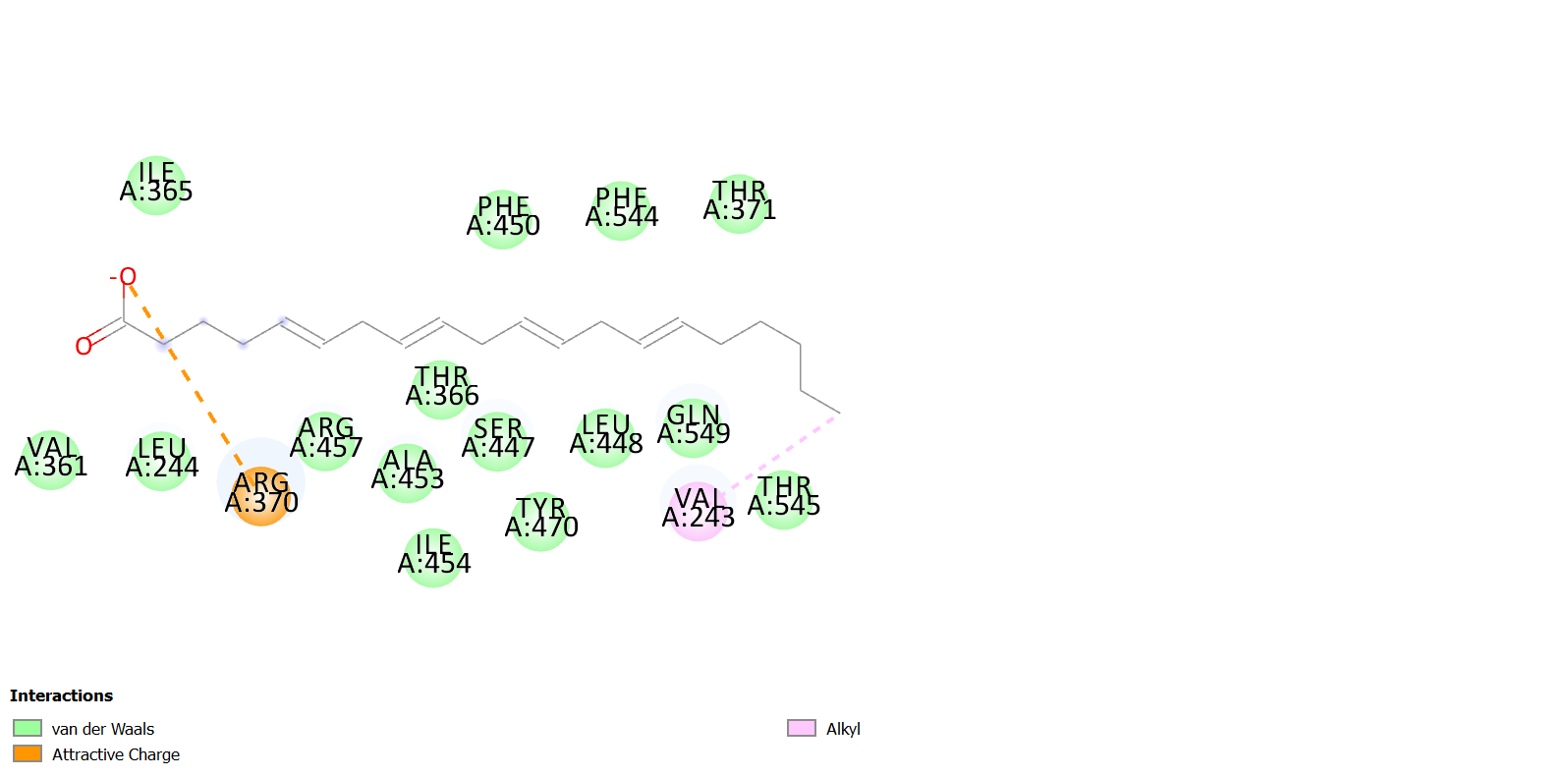

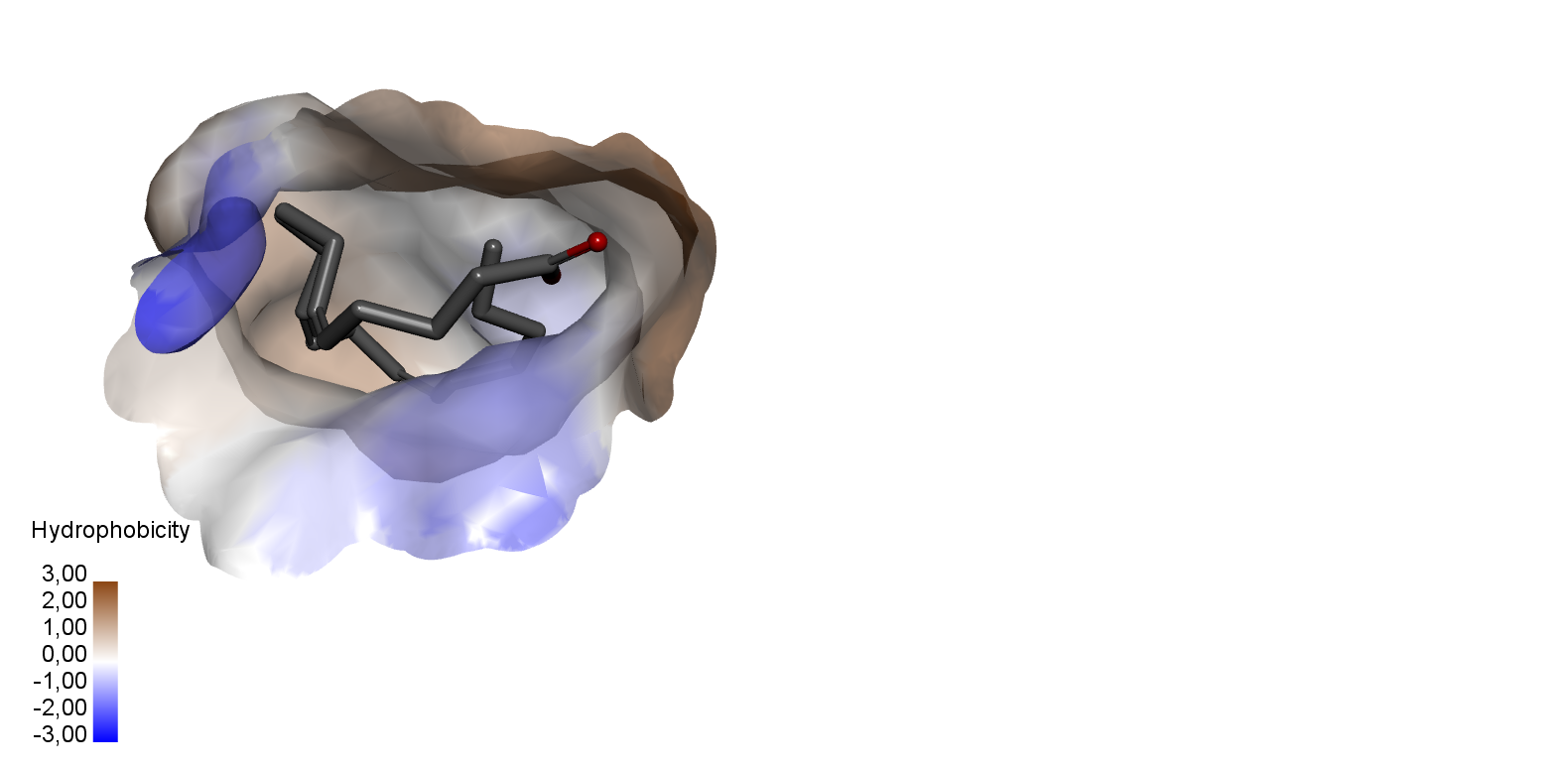


**A**

**C**

**B**

**D**
